# Variability and effectiveness of comparator group interventions in smoking cessation trials: a systematic review and meta‐analysis

**DOI:** 10.1111/add.14969

**Published:** 2020-02-11

**Authors:** Nicola Black, Maarten C. Eisma, Wolfgang Viechtbauer, Marie Johnston, Robert West, Jamie Hartmann‐Boyce, Susan Michie, Marijn de Bruin

**Affiliations:** ^1^ Health Psychology Group, Institute of Applied Health Sciences University of Aberdeen, Health Sciences Building, Foresterhill Aberdeen UK; ^2^ National Drug and Alcohol Research Centre University of New South Wales Sydney NSW Australia; ^3^ Department of Clinical Psychology and Experimental Psychopathology University of Groningen Groningen the Netherlands; ^4^ Department of Psychiatry and Neuropsychology, School for Mental Health and Neuroscience Maastricht University Maastricht the Netherlands; ^5^ Department of Behavioral Science and Health University College London London UK; ^6^ Nuffield Department of Primary Care Health Sciences Oxford UK; ^7^ Centre for behavior Change University College London Gower Street London WC1E 6BT UK; ^8^ Radboud University Medical Center Radboud Institute for Health Sciences, IQ Healthcare Nijmegen the Netherlands

**Keywords:** Behavior change techniques, comparator group, control group, meta‐analysis, meta‐regression, smoking cessation, systematic review

## Abstract

**Aims:**

To examine variability and effectiveness of interventions provided to comparator (control) groups in smoking cessation trials.

**Methods:**

Systematic review with meta‐analysis of randomized controlled trials (RCTs) of behavioral interventions for smoking cessation, with or without stop‐smoking medication. We searched the Cochrane Tobacco Addiction Group Specialized Register for RCTs with objective outcomes measured at ≥ 6 months. Study authors were contacted to obtain comprehensive descriptions of their comparator interventions. Meta‐regression analyses examined the relationships of smoking cessation rates with stop‐smoking medication and behavior change techniques.

**Results:**

One hundred and four of 142 eligible comparator groups (*n* = 23 706) had complete data and were included in analyses. There was considerable variability in the number of behavior change techniques delivered [mean = 15.97, standard deviation (SD) = 13.54, range = 0–45] and the provision of smoking cessation medication (43% of groups received medication) throughout and within categories of comparator groups (e.g. usual care, brief advice). Higher smoking cessation rates were predicted by provision of medication [*B* = 0.334, 95% confidence interval (CI) = 0.030–0.638, *P* = 0.031] and number of behavior change techniques included (*B* = 0.020, 95% CI = 0.008–0.032, *P* < 0.001). Modelled cessation rates in comparator groups that received the most intensive support were 15 percentage points higher than those that received the least (23 versus 8%).

**Conclusions:**

Interventions delivered to comparator groups in smoking cessation randomized controlled trials vary considerably in content, and cessation rates are strongly predicted by stop‐smoking medication and number of behavior change techniques delivered.

## Introduction

Tobacco smoking is a leading cause of premature mortality, disease and health‐care expenditures 1, 2. Numerous smoking cessation interventions have been developed and their evidence synthesized in systematic reviews, including multiple Cochrane reviews 3, 4, 5, 6, 7, 8, 9, 10, 11. The evidence generated has informed smoking cessation guidelines and health‐care services and helped numerous people to quit smoking 12, 13. Accurate evidence can optimize the effectiveness of these interventions and ensure that services offered are those which are most cost‐effective.

The effectiveness of smoking cessation interventions is commonly determined by comparing them with an active control. As such, the observed effect sizes will be a function of the effectiveness of the intervention provided to the experimental group, but also the intervention provided to the corresponding control group, or ‘comparator group’, 1We use the term ‘comparator group’ in recognition of the fact that many of these groups receive (comparator) interventions. as well as of other variables. Study aims vary from trial to trial, and the type of comparator employed should covary with these aims 14. At the most minimal, comparator groups receive no support and simply report their outcomes (which, arguably, could be considered very brief support in itself) 15. At the most intensive, comparator groups might receive many counselling sessions. Systematic reviews of behavioral interventions tend to either ignore the variability in comparator interventions or try to address it by organizing comparator groups into broad categories, such as no behavioral support, self‐help [e.g. pamphlets, self‐directed workbooks, applications (apps)], brief advice/counselling (e.g. a short, typically less than 30 minutes, amount of advice/counselling), extended counselling (longer, often multiple sessions, of counselling), usual care (any support already typically delivered in practice) and/or some combination of these categories 3, 4, 5, 6, 7, 8. This approach might not fully account for the variability between comparator groups, as evidence from other areas shows that intervention content delivered to ‘usual care’ groups can vary considerably between trials 16. In a previous systematic review of HIV medication adherence interventions, this variability in usual care content explained up to 34% points differences in the clinical outcomes observed in the comparator groups and influenced trial effect sizes 16, 17. This suggests that, without accounting for the differences in the intervention content provided to comparator groups, it might not be possible to directly synthesize experimental intervention effects from these trials. Further, for readers of these trials—such as those assessing whether a given intervention will improve outcomes over their current practice—it might also not be possible to interpret, compare or generalize the results.

The present aim was to examine the variability and effectiveness of comparator intervention content in a much larger and more heterogeneous body of randomized controlled trials (RCTs) of behavioral interventions for smoking cessation among adults 18. Based on previous research, our pre‐registered (https://osf.io/24pzj/) primary hypotheses were that provision of pharmacological support (i.e. smoking cessation medication) and behavioral support [i.e. higher number of smoking cessation behavior change techniques (BCTs)] 19, 20 would vary between comparator groups and predict higher smoking cessation. We also predicted that BCTs delivered in a personalized manner would be more effective than those delivered in a non‐personalized manner (i.e. a one‐size‐fits‐all approach). Secondary hypotheses were that behavioral support delivered by a person would be more effective than support delivered in writing (digital and/or print), that more adjuvant support to engage participants with the intervention would predict higher cessation rates and that, among those who received medication, more adjuvant support to increase medication adherence would predict higher cessation rates.

## Methods

### Reporting standards

This study is part of a larger systematic review of behavioral smoking cessation trials IC‐SMOKE; 18. The project is registered on the International Prospective Register of Systematic Reviews (PROSPERO) (CRD42015025251) and the Open Science Framework (https://osf.io/23hfv/). The completed Preferred Reporting Items for Systematic Reviews and Meta‐Analyses (PRISMA) checklist is in Supporting information, Appendix A 21.

### Eligibility

The Cochrane Tobacco Addiction Group Specialized Register was searched on 1 November 2015 for RCTs assessing the impact of behavioral interventions (with or without smoking cessation medication) on biochemically verified smoking cessation at 6 months or longer. Trials without biochemically verified outcomes were excluded to protect against multiple sources of bias 22, 23. Trials were excluded if they were published before 1996, were not reported in English (as resources for translation of documentation were not available) or in peer‐reviewed journals, or if participants were aged under 18 years. Trials published before 1996 were excluded because older trials of behavioral interventions are less relevant in a continually changing social and policy environment, and because preliminary work indicated that it was very difficult to retrieve the required materials from authors of trials published beyond 20 years earlier. The comparator groups included in the present analyses were the single least intensive groups in each RCT (i.e. one comparator group per RCT), which could have included no support, medication, usual care or comparator interventions introduced by the researchers. Detailed methods are described in the Intervention and Comparison group support provided in SMOKing cEssation (IC‐SMOKE) protocol 18.

### Procedure

Data were first extracted from published materials (e.g. primary articles, appendices, protocols, intervention development papers). A comprehensive procedure of contacting authors of all included trials was then executed to retrieve additional, unpublished materials 24. First/corresponding authors were contacted by e‐mail (including several reminders), followed‐up by telephone as required. If the first/corresponding author did not reply or was unable to help, the second/last authors were contacted, followed by middle authors, as required. Authors were asked to provide additional materials on the intervention provided to their comparator group (e.g. manuals, practitioner training materials, self‐help materials, website content) and to complete a comparator intervention checklist. The comparator intervention checklist (https://osf.io/e834t/) was a purpose‐built questionnaire capturing smoking cessation activities (Javornik *et al.,* unpublished). We developed it based on international stop smoking treatment manuals, input from advisory board members (of smokers/ex‐smokers, smoking cessation professionals and policymakers), expertise within the study team and smoking cessation examples provided in previous BCT taxonomies. A similar approach was shown to be reliable and valid in the previous HIV medication adherence systematic review 16.

The active content provided to comparator groups (namely, BCTs and smoking cessation medications) was extracted from the above‐described materials. Two researchers independently and reliably 25 used the BCT taxonomy (BCTTv1; Supporting information, Appendix A) 19—with one BCT added and one BCT removed—to code the presence/absence of 93 individual BCTs, the behavior targeted by each BCT (smoking cessation behaviors: making a quit attempt, remaining abstinent; adjuvant behaviors: adhering to medication, engaging in treatment) and whether the BCT delivery was personalized (i.e. individually tailored or requiring active recipient involvement). Examples of BCTs are reducing prompts or cues that might trigger smoking, considering the pros and cons of quitting and verbally persuading the person that they are capable of quitting. Following retrieval and coding of all available materials, the extent to which these materials comprehensively described the active intervention content was determined using independent double coding [see Supporting information, Appendix A for the decision model used; prevalence and bias‐adjusted kappa (PABAK) = 0.79]. The comparator interventions were labelled as well described if the coders judged the materials to be of sufficient detail and clarity to identify all or almost all the BCTs that were delivered to that comparator group. Finally, comparators were also coded as to whether the BCTs were primarily delivered in writing (digital/print) or by a person (face‐to‐face or via telephone).

### Data analysis

The a priori prepared analysis plan was published on the Open Science Framework before conducting the analyses (https://osf.io/23hfv/). Analyses were conducted using the metafor package in R 26. The analysis script and data (https://osf.io/gk56j/) are also available on the Open Science Framework. Multi‐level, mixed‐effects meta‐regression models were used to examine the association between comparator intervention content and (logit‐transformed) smoking cessation rates. Outcome time points were all those at 6 months (± 1 month) 2To allow for variation between studies in the exact time at which follow‐up assessments were conducted, we allowed all time‐points at 5 months or later to be included. post‐randomization or later (i.e. multiple time‐points per study were permitted). Multiple outcome time‐points were included, as this provides a more thorough synthesis of the available evidence than would be afforded by omitting all data other than those of a single time‐point. The model included random intercepts for studies (to account for between‐study heterogeneity), 3As each study contributed a single comparator group to the analyses, study and group are synonymous in this context. correlated random effects for multiple observations (i.e. logit rates) within studies with a continuous time autoregressive structure (to account for heterogeneity in multiple observations corresponding to the same group) and correlated sampling errors for multiple observations within studies (to account for the dependency between multiple observations corresponding to the same group). For the sampling errors, we conservatively assumed an autocorrelation coefficient of ρ = 0.9 for a lag of 1 month. The smoking cessation rates extracted from the studies were based on intent‐to‐treat analyses with missing responses treated as smokers (assumption: missing = smoking; 27). Only those comparator groups rated as well described were included in the primary analyses. Various sensitivity analyses were conducted, including fitting the models while controlling for attrition, including all comparator groups, and using robust variance estimation 28. These are described in full in the analysis plan.

In the first primary model, smoking cessation rates were regressed on the degree of behavioral support (number of smoking cessation BCTs; i.e. those targeting quitting or abstinence) and the provision of medication (0 = no, 1 = yes; model 1). In the second primary model, smoking cessation rates were regressed on the number of personalized BCTs, the number of non‐personalized BCTs and the provision of medication (model 2).

In the secondary analyses, we first examined (models 3 and 4) whether the effect of BCTs on smoking cessation depends on the mode of delivery [i.e. whether comparator interventions were primarily delivered in writing (= 0) or by a person (= 1)] by adding a mode of delivery main effect and its interaction with smoking cessation BCTs to models 1 and 2. To investigate the additional value of supporting the adjuvant behaviors (models 5 and 6), we added number of treatment engagement BCTs, number of medication adherence BCTs and the interaction between medication provision and number of medication adherence BCTs (as adherence BCTs should primarily benefit people who were provided with medication) to models 1 and 2, respectively. Exploratory analyses were also conducted; namely, to investigate potential differences over time, differences by quitting versus abstinence BCTs, and interactions between smoking cessation BCTs and medication in determining smoking cessation. The results of these are described in Supporting information, Appendix B.

All analyses were controlled for potential confounding variables that we identified through literature review and input from our advisory board panel 29, 30, 31. These control variables were: (1) mean age (in years), (2) mean nicotine dependence (using the Fagerström Test for Nicotine Dependence; theoretical range: 0–10; missing values imputed based on cigarettes per day, where available 32), (3) length of follow‐up (coded in 6‐month units to facilitate interpretation; log‐transformed to pre‐emptively avoid any undue influence of a few very long follow‐up assessments), (4) cotinine verification [1 = yes, 0 = no, where no includes less stringent biochemical verification types, such as exhaled carbon monoxide (CO)] and (5) type of abstinence assessed (1 = sustained, 0 = point prevalence). The selection of these variables is discussed further in the analysis plan.

### Future research

Smoking is a leading cause of premature mortality, disease and health‐care expenditures 1, 2, and the current study suggests that existing smoking cessation intervention research might be overlooking an important source of clinical, and possibly statistical, heterogeneity. We are currently conducting analyses to determine if and how the observed variability in comparator groups impacts conclusions about the relative effectiveness of different types of interventions, with the goal of producing more accurate estimates of intervention effects (https://osf.io/khm8u/). Further, as the link between comparator interventions and outcomes has now been established in both the HIV medication adherence 16, 17 and smoking cessation domains, it will be important to investigate whether similar effects are occurring in other domains of behavioral interventions, and complex interventions more generally. To enable this, it is essential that trial authors provide detailed descriptions of their comparator interventions, as published descriptions are often incomplete 24, 33, 34, 35, 36. New tools such as the *Addiction* Journal's Paper Authoring Tool might facilitate this 37.

The results of this study highlight avenues for research into improving usual care for smoking cessation. Researchers could investigate whether adding additional BCTs—especially BCTs delivered by a person—into existing programmes leads to higher rates of smoking cessation. Further, to operationalize comparator group support we took a pragmatic approach using a simple sum score of BCTs. To provide better guidance on which support is most effective, it will be important to investigate which combinations of BCTs are associated with the highest smoking cessation rates (as we are currently doing: https://osf.io/m5vea/). Finally, it will be important to investigate how BCTs can be best translated to be delivered in writing (digital and/or print), as we did not find evidence of an association with smoking cessation when delivered through these mediums. At present, such interventions produce small increases in smoking cessation 9, 10 and identifying which components of these are most effective could help us increase the effectiveness of these interventions.

## Results

### Study identification

**Figure 1:**
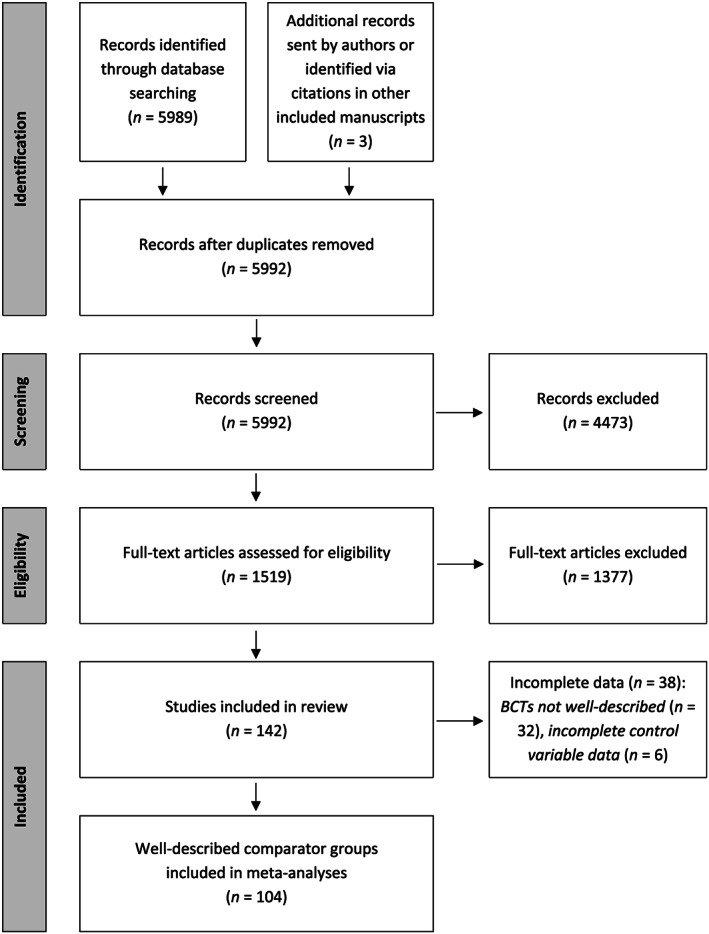
Preferred Reporting Items for Systematic Reviews and Meta‐Analyses (PRISMA) flow diagram. BCTs = behavior change techniques

Initially, 5992 records were identified (Fig. 1). Following screening and eligibility assessment, 142 unique trials were included (see Supporting information, Appendix B for a list of included studies); 110 of 142 (77%) comparator groups were rated as well described, after retrieving additional information from authors on 93 of 142 (65%) of comparator groups. Of the 110 well‐described comparator groups, complete data on all primary predictor and control variables were available for 104 groups, and these are analysed here. This included *n* = 23 706 participants and 161 time‐points ranging from 22 to 130 weeks post‐randomization (one study contributed five time‐points, three contributed four time‐points, six contributed three time‐points, 32 contributed two time‐points and the remaining 62 contributed a single time‐point).

### Variability in behavioral and pharmacological support

There was considerable variability between the 104 comparator groups in the number of smoking cessation BCTs delivered (mean = 15.97, SD = 13.54, range = 0–45), number of personalized smoking cessation BCTs delivered (mean = 3.12, SD = 3.93, range = 0–16) and provision of medication (43%). The most commonly delivered smoking cessation BCTs were unspecified social support, tell to act and information about health consequences (delivered to 81, 67 and 67% of comparator groups, respectively). The most commonly delivered personalized smoking cessation BCTs were unspecified social support, behavioral goal‐setting and reducing exposure to cues (delivered to 50, 27 and 23% of comparator groups, respectively).

Further, when grouping the comparators in categories typically used in systematic reviews of smoking cessation trials, variability in the number of BCTs delivered to ‘no behavioral support’ comparator groups was low (mean = 1.38, SD = 2.48, range = 0–9, *k* = 21), but considerable variability remained within the other categories: ‘self‐help’ (mean = 18.00, SD = 8.54, range = 5–32, *k* = 10), ‘brief advice’ (mean = 12.11, SD = 11.08, range = 0–45, *k* = 37), ‘extended counselling’ (mean = 27.89, SD = 9.99, range = 5–43, *k* = 36) and ‘usual care’ (mean = 12.07, SD = 13.50, range = 0–45, *k* = 28 [5]). Hence, even within these typical categories, some comparator groups receive little to no support, whereas others receive extensive support.

### Association between intervention active content and smoking cessation

Provision of smoking cessation medication and more smoking cessation BCTs predicted higher smoking cessation rates (model 1, Table 1). The BCT‐smoking cessation association seemed to be driven both by personalized BCTs and non‐personalized BCTs, as these associations with cessation rates were not significantly different (*P* = 0.400; model 2). Given that these two predictors were correlated (*r* = 0.49, *P* < 0.001), we re‐ran the models with each predictor separately, which gave slightly larger differences in effect sizes and smaller *P*‐values (personalized: *B* = 0.057, *P* = 0.004; non‐personalized: *B* = 0.022, *P* = 0.002), suggesting that both variables were competing for the same variance. These results suggest that delivering more personalized and non‐personalized BCTs, as well as smoking cessation medication, predicts higher smoking cessation rates in these comparator groups.

**Table 1:** Meta‐regression results [B (SE)] predicting (logit‐transformed) comparator group smoking cessation rates from active content provided.

	Primary models		Secondary models			
	Model 1	Model 2	Model 3	Model 4	Model 5	Model 6
Predictors						
Received medication (1 = yes, 0 = no)	0.334 (0.155)*	0.346 (0.156)*	0.266 (0.166)	0.231 (0.164)	0.429 (0.251)^a^, †	0.399 (0.260)^a^
Total smoking cessation BCTs	0.020 (0.006)***		−0.020 (0.027)^a^		0.010 (0.008)	
Personalized smoking cessation BCTs		0.038 (0.022)†		−0.131 (0.068)^a^, †		0.021 (0.023)
Non‐personalized smoking cessation BCTs		0.016 (0.008)*		0.031 (0.038)^a^		0.007 (0.010)
Mode of delivery (1 = person‐delivered, 0 = written)			−0.605 (0.546)^a^	−0.235 (0.564)^a^		
Total smoking cessation BCTs × mode of delivery			0.042 (0.028)			
Personalized smoking cessation BCTs × mode of delivery				0.186 (0.071)**, b		
Non‐personalized smoking cessation BCTs × mode of delivery				−0.016 (0.039)		
Treatment engagement BCTs					0.176 (0.079)*, c	0.171 (0.080)*, c
Medication adherence BCTs					0.044 (0.078)^a^	0.046 (0.078)^a^
Medication adherence BCTs × received medication					−0.067 (0.089)	−0.057 (0.092)
Model						
Groups	104	104	88^d^	88^d^	104	104
Time‐points	161	161	142^d^	142^d^	161	161

a Variables that formed part of interaction terms were not mean‐centred. The ‘main’ effects of these variables should be interpreted as the effect of that variable when the other from the interaction term = 0; e.g. in model 3, the ‘main’ effect of BCTs is the simple effect when mode of delivery is written (mode of delivery = 0) and the ‘main’ effect of mode of delivery is the simple effect when no BCTs were delivered (BCTs = 0).

b Removal of one influential case resulted in the interaction term becoming non‐significant (*B* = 0.145, *P* = 0.102) and cluster robust re‐estimation resulted in this effect becoming significant again (*B* = 0.145, *P* = 0.003).

c Removal of one influential case resulted in the effect of treatment engagement BCTs becoming non‐significant (5: *B* = 0.182, *P* = 0.081; 6: *B* = 0.170, *P* = 0.115) and this remained non‐significant following cluster robust re‐estimation (5: *B* = 0.182, *P* = 0.074; 6: *B* = 0.170, *P* = 0.097).

d Sample size reduced because model includes only active comparator groups (passive comparator groups do not have a mode of delivery).

BCT = behavior change technique; SE = standard error. All models were controlled for mean age, mean Fagerström Test for Nicotine Dependence, length of follow‐up, cotinine verification and point prevalence versus sustained abstinence.

*** *P* < 0.001;

** *P* < 0.01;

* *P* < 0.05;

† *P* < 0.1.

### Interaction between mode of delivery and BCTs in predicting smoking cessation

Whether smoking cessation BCTs are more strongly predictive of smoking cessation when delivered by a person than when delivered in writing (digital and/or print) was tested in models 3 and 4. The association between smoking cessation rates and number of BCTs delivered was evident for those BCTs delivered by a person (*B* = 0.022, *P* = 0.001), but not those delivered in writing (*B* = −0.020, *P* = 0.452); although the direct interaction test did not suggest that these association differed (model 3).

When BCTs were personalized, the association with higher smoking cessation was again evident when they were delivered by a person (*B* = 0.055, *P* = 0.013), but not when delivered in writing (*B* = −0.131, *P* = 0.052), and these associations were significantly different (model 4). There was no evidence that non‐personalized BCTs delivered in writing (*B* = 0.031, *P* = 0.416) or by a person (*B* = 0.015, *P* = 0.072) predicted smoking cessation. Together, these results show that personalized and person‐delivered BCTs are associated with higher smoking cessation, with no support found for non‐personalized BCTs or those delivered in writing.

### Association between BCTs targeting adjuvant behaviors and smoking cessation

The associations between BCTs targeting adjuvant behaviors and smoking cessation rates were tested in models 5 and 6. Higher smoking cessation rates were predicted by provision of more BCTs to engage the participant in the treatment (models 5 and 6), but not by more BCTs to aid medication adherence (model 5: *B* = −0.022, *P* = 0.698; model 6: *B* = −0.011, *P* = 0.861 among those groups who were provided medication). The association between number of treatment engagement BCTs and smoking cessation was attenuated by the removal of one influential case (model 5: *B* = 0.182, *P* = 0.081; model 6: *B* = 0.170, *P* = 0.115). The comparator group in this study 38 received a large number of treatment engagement BCTs relative to all other comparator groups (8 versus range of others: 0–3), and this seemed to determine the results of this analysis.

### Estimated smoking cessation rates at different levels of behavioral and pharmacological support

**Figure 2:**
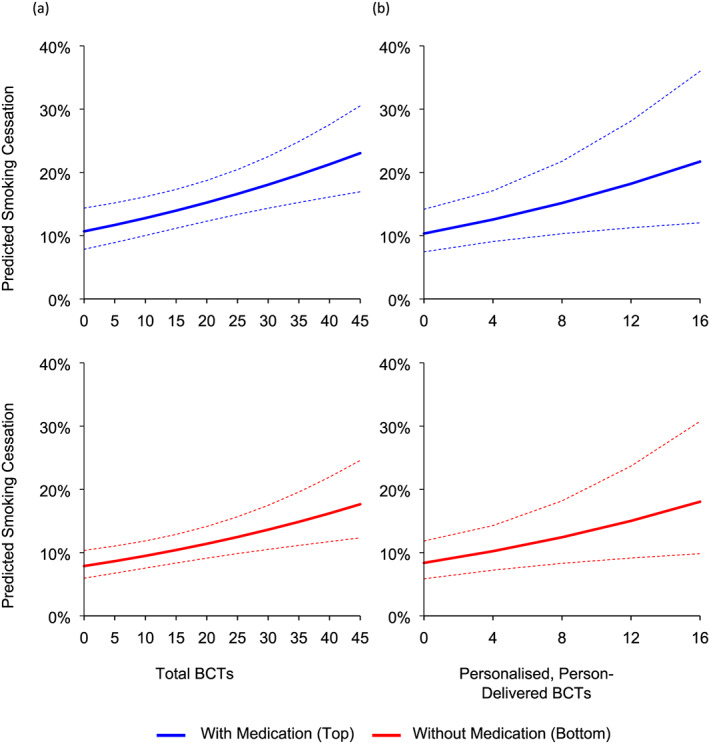
Predicted smoking cessation rates across the observed range of (a) total behavior change techniques (BCTs) and (b) personalized, person‐delivered BCTs. Dotted lines represent 95% confidence intervals. Estimated smoking cessation rates are computed at 6‐month follow‐up, non‐cotinine verified, point prevalence abstinence and mean levels of age and nicotine dependence. [Colour figure can be viewed at wileyonlinelibrary.com]

The predicted impact of varying levels of behavioral and pharmacological support on smoking cessation rates is shown in Fig. 2. As seen, our models predicted that, on average, 8% of those who received no behavioral or pharmacological support will be abstinent. If participants received 45 BCTs (the observed maximum), then the predicted cessation rate increased to 18%. If participants also received smoking cessation medication, this rate increased to 23%. Similarly, if participants received 16 personalized, person‐delivered BCTs (the observed maximum), the predicted cessation rate was 18% without medication and 22% with medication. Note the different scales on the *x*‐axes in Fig. 2a versus Fig. 2b, illustrating that similar smoking cessation rates might be achieved using fewer BCTs if those BCTs are personalized and delivered by a person.

## Discussion

### Principal findings

This study examined the variability in, and effectiveness of, the active content of interventions (namely, BCTs and smoking cessation medications) provided to comparator groups in RCTs of behavioral interventions for smoking cessation. The active content varied considerably among all comparator groups and within typical categories of comparator groups (e.g. usual care, brief advice). Further, delivery of smoking cessation medication and more behavioral support predicted higher smoking cessation rates in these comparator groups. The predicted effect of this variability was a 15%‐point absolute difference in cessation rates between comparator groups, with the least and most intensive comparator interventions predicting 8 and 23% cessation, respectively. This difference between comparator groups is greater than the typical differences seen between experimental and comparator groups in smoking cessation trials 4, 5, 39.

This study replicates and extends earlier work, in which the authors raised the issue of variability in interventions delivered to comparator groups of behavioral trials and its implications for interpreting and comparing effect sizes 16, 17. This work was performed in the area of HIV medication adherence interventions in a small set of only usual care comparator groups. That these results have now also been obtained for a substance use/addictive behavior (smoking) in a large set of studies with multiple categories of comparator groups clearly supports the idea that readers and systematic reviewers need to consider variability in comparator interventions when interpreting, comparing and generalizing trial effect sizes 17, 40.

This study also found support for the potential role of BCTs in increasing smoking cessation rates, particularly when personalized and delivered by a person, and did not find evidence that the effect of these BCTs on cessation declines over time (Supporting information, Appendix B). This adds to existing literature, which has shown stronger intervention effects when interventions are delivered, at least in part, by a person (compared to self‐help alone) and when interventions are tailored to the participant 5, 41, 42, 43. This study also adds to the mixed, broader health behavior change literature, which has found some positive relationships between the number of BCTs used and smoking cessation 44 and other behaviors (e.g. 45, 46, 47) but which, overall, typically finds non‐significant relationships with smoking cessation 48 and other outcomes (e.g. 36, 49, 50, 51, 52, 53, 54, 55, 56, 57). The strengths of our methodology (retrieval of unpublished materials, restriction of analyses to well‐described studies) might have increased the accuracy of our BCT data. Along with the much larger number of included studies, this would increase power to detect the BCTs–cessation relationship.

### Strengths and limitations

The key strengths of this study are, first, the retrieval of extensive, unpublished materials describing comparator interventions from authors and the restriction of analyses to well‐described comparators. Secondly, the inclusion of a priori specified confounders and the observed dose–response relationship increase confidence in a potential causal link between comparator interventions and outcomes 58. The key limitation is that analyses were correlational and unaddressed confounders could be driving the associations. Other intervention factors, such as frequency or duration of interpersonal contact, could influence outcomes. Nonetheless, if this is the case, the implication that variability in comparator interventions warrants consideration remains unchanged. Further, we included only English language publications, meaning that our review does not cover comparator groups from RCTs published exclusively in other languages. Finally, our BCT variable was a sum score, which therefore assumed that all BCTs are equally effective; this may not be the case. However, in the absence of evidence of the effectiveness of each BCT in this context, this pragmatic approach was judged to be most suitable.

### Implications for policy and practice

Policymakers and practitioners use publications about trials and systematic reviews to evaluate which interventions to fund and implement. Our results indicate that, when doing so, it is key to consider against what comparators these interventions have been tested. We observed large (15% points) predicted differences in smoking cessation between comparator groups that were associated with the level of support received. This variability could make some experimental interventions appear much stronger than others whereas, in fact, this might have nothing to do with the experimental interventions; instead, it might be due to one trial having a minimal and another trial an intensive comparator intervention. Currently used methods for accounting for comparator group variability (i.e. separating meta‐analyses by categories of comparator groups) may go some distance, but as variability in active content within commonly used categories is substantial this is unlikely to fully address the issue. Hence, we recommend that researchers, policymakers and practitioners ensure that full information on the comparator interventions is available and considered when interpreting, comparing and generalizing intervention effects and when making decisions on which services to fund and implement.

## Conclusions

Interventions provided to comparator groups in smoking cessation trials vary substantially and predict cessation rates in these groups. This variability should be considered when synthesizing, interpreting, comparing or generalizing intervention effects.

## Systematic review registration

PROSPERO (CRD42015025251) and the Open Science Framework (analysis plan: https://osf.io/24pzj/).

## Declaration of interests

R.W. undertakes research and consultancy for companies that develop and manufacture smoking cessation medications (Pfizer, J&J and GSK). R.W. is an unpaid adviser to the UK's National Centre for Smoking Cessation and Training. R.W.’s salary is funded by Cancer Research UK. N.B. and M.C.E.’s salaries were funded by Cancer Research UK. J.H.B. receives funding from the National Institute for Health Research (NIHR) Oxford Biomedical Research Centre (BRC). The views expressed are those of the author(s) and not necessarily those of the NHS, the NIHR or the Department of Health and Social Care. All other authors declare that they have no conflicts of interest. The National Drug and Alcohol Research Centre is supported by funding from the Australian Government Department of Health under the Drug and Alcohol Program.

## Supporting information


**Data S1**. Supporting information
