# Supplementary material for: Variability and effectiveness of comparator group interventions in smoking cessation trials: a systematic review and meta‐analysis
Source: Addiction. 2020 Feb 11;115(9):1607–17. doi: 10.1111/add.14969 (PMC7496125; doi:10.1111/add.14969)
Supplement: Supplementary file 1 — Data S1. Supporting information [file ADD-115-1607-s001.docx]

**APPENDIX FOR:**

**Variability and effectiveness of comparator group interventions in smoking cessation trials: A systematic review and meta-analysis**

Black, Eisma, Viechtbauer, Johnston, West, Hartmann-Boyce, Michie, and de Bruin

**Contents**

[Appendix A: Additional methodological information 2](#_Toc20991525)

[Adapted Behaviour Change Technique Taxonomy Version 1 used in the Current Study 2](#_Toc20991526)

[Decision Tree used to Rate the Quality of Comparator (and Experimental) Group Intervention Descriptions 26](#_Toc20991527)

[Preferred Reporting Items for Systematic Reviews and Meta-Analyses (PRISMA) Checklist 27](#_Toc20991528)

[Appendix B: Additional results information 29](#_Toc20991529)

[Studies Included in this Systematic Review 29](#_Toc20991530)

[Exploratory and Sensitivity Analyses 37](#_Toc20991531)

[Interactions Effects by Time and BCT Target on Smoking Cessation Rates 37](#_Toc20991532)

[Predicting Smoking Cessation while including all p < .1 Predictors 38](#_Toc20991533)

[Predicting Smoking Cessation while including All^a^ Studies and Controlling for Attrition 39](#_Toc20991534)

[Interactions Effects by Smoking Cessation BCTs and Receipt of Medication on Smoking Cessation Rates 42](#_Toc20991535)

Appendix A: Additional methodological information

**Supplemental Table A1.**

Adapted Behaviour Change Technique Taxonomy Version 1 used in the Current Study

| **BCT** | **BCTTv1 Example** | **Smoking Example** |
| --- | --- | --- |
| **1. Goals and planning** | | |
| - 1. **Goal setting (behaviour)**   Set or agree on a goal defined in terms of the behaviour to be achieved  *Note: only code goal-setting if there is sufficient evidence that goal set as part of intervention;* *if goal unspecified or a behavioural outcome, code* ***1.3, Goal setting (outcome)****; if the goal defines a specific context, frequency, duration or intensity for the behaviour, also code* ***1.4, Action planning*** | Agree on a daily walking goal (e.g. 3 miles) with the person and reach agreement about the goal  Set the goal of eating 5 pieces of fruit per day as specified in public health guidelines | Agree on a quit date with the client |
| **1.2 Problem solving**  Analyse, or prompt the person to analyse, factors influencing the behaviour and generate or select strategies that include overcoming barriers and/or increasing facilitators (includes ‘**Relapse Prevention***’ and ‘***Coping Planning***’*)  *Note: barrier identification without solutions is not sufficient. If the BCT does not include analysing the behavioural problem, consider* ***12.3****,* ***Avoidance/changing exposure to cues for the behaviour, 12.1, Restructuring the physical environment,*** ***12.2,*** ***Restructuring the social environment,*** *or* ***11.2, Reduce negative emotions*** | Identify specific triggers (e.g. being in a pub, feeling anxious) that generate the urge/want/need to drink and develop strategies for avoiding environmental triggers or for managing negative emotions, such as anxiety, that motivate drinking  Prompt the patient to identify barriers preventing them from starting a new exercise regime e.g., lack of motivation, and discuss ways in which they could help overcome them e.g., going to the gym with a buddy | Identify specific triggers (e.g. being in a pub, feeling anxious) that generate the urge/want/need to smoke and develop strategies for avoiding environmental triggers that motivate smoking |
| **1.3 Goal setting (outcome)**  Set or agree on a goal defined in terms of a positive **outcome** of wanted behaviour  *Note:* *only code guidelines if set as a goal in an intervention context; if goal is a behaviour, code* ***1.1, Goal setting (behaviour)****; if goal unspecified code* ***1.3, Goal setting (outcome)*** | Set a weight loss goal (e.g. 0.5 kilogram over one week) as an outcome of changed eating patterns | Set a goal with the client to have a CO-level of a non-smoker for the next meeting |
| **1.4 Action planning**  Prompt detailed planning of performance of the behaviour (must include at least one of context, frequency, duration and intensity). Context may be environmental (physical or social) or internal (physical, emotional or cognitive) (includes *‘***Implementation Intentions***’*)  *Note: evidence of action planning does not necessarily imply goal setting, only code latter if sufficient evidence* | Encourage a plan to carry condoms when going out socially at weekends  Prompt planning the performance of a particular physical activity (e.g. running) at a particular time (e.g. before work) on certain days of the week | Prompt planning of preparatory behaviour to facilitate quitting smoking (e.g. remove tobacco products from the house, tell others about the quit attempt) on specific days during the coming week  Prompt the client to make a plan how, when and where medication will be used. |
| **1.5 Review behaviour goals**  Review behaviour goal(s) jointly with the person and consider modifying goal(s) or behaviour change strategy in light of achievement. This may lead to re-setting the same goal, a small change in that goal or setting a new goal instead of (or in addition to) the first, or no change *Note:* *if goal specified in terms of behaviour, code* ***1.5, Review behaviour goal(s)****, if goal unspecified, code* ***1.7, Review outcome goal(s);*** *if* *discrepancy created consider also* ***1.6, Discrepancy between current behaviour and goal*** | Examine how well a person’s performance corresponds to agreed goals e.g. whether they consumed less than one unit of alcohol per day, and consider modifying future behavioural goals accordingly e.g. by increasing or decreasing alcohol target or changing type of alcohol consumed | Review with the client if the main goal of abstinence from cigarettes has been achieved. This may lead to resetting a quit date if the client has lapsed. |
| **1.6 Discrepancy between current behaviour and goal**  Draw attention to discrepancies between a person’s current behaviour (in terms of the *form, frequency, duration, or intensity* of that behaviour) and the person’s previously set outcome goals, behavioural goals or action plans (goes beyond self-monitoring of behaviour)  *Note: if discomfort is created only code* ***13.3, Incompatible beliefs*** *and not* ***1.6, Discrepancy between current behaviour and goal****;* *if goals are modified, also code* ***1.5, Review behaviour goal(s)*** *and/or* ***1.7, Review outcome goal(s)****; if feedback is provided, also code* ***2.2, Feedback on behaviour*** | Point out that the recorded exercise fell short of the goal set | Draw attention to the fact that the client has not met the goal of not smoking after the quit date. |
| **1.7 Review outcome goal**  Review outcome goal(s) jointly with the person and consider modifying goal(s) in light of achievement. This may lead to re-setting the same goal, a small change in that goal or setting a new goal instead of, or in addition to the first  *Note:* *if goal specified in terms of behaviour, code* ***1.5, Review behaviour goal(s)****, if goal unspecified, code* ***1.7, Review outcome goal(s);*** *if* *discrepancy created consider also* ***1.6, Discrepancy between current behaviour and goal*** | Examine how much weight has been lost and consider modifying outcome goal(s) accordingly e.g., by increasing or decreasing subsequent weight loss targets | Review with the client whether the goal set for CO-reading has been met. This may lead to re-setting the same goal with regard to CO-levels or a small change in the goal on CO-levels. |
| **1.8 Behavioural contract**  Create a written specification of the behaviour to be performed, agreed on by the person, and witnessed by another  *Note:* *also code* ***1.1, Goal setting (behaviour)*** | Sign a contract with the person e.g. specifying that they will not drink alcohol for one week | Sign a written contract with the client stating that the client will not smoke after their quit date |
| **1.9 Commitment**  Ask the person to affirm or reaffirm statements indicating commitment to change the behaviour  *Note:* *if defined in terms of the behaviour to be achieved also code* ***1.1, Goal setting (behaviour)*** | Ask the person to use an “I will” statement to affirm or reaffirm a strong commitment (i.e. using the words “strongly”, “committed” or “high priority”) to start, continue or restart the attempt to take medication as prescribed | Ask the person to use an “I will” statement to affirm or reaffirm a strong commitment (i.e. using the words “strongly”, “committed” or “high priority”) to quit smoking. |
| **2. Feedback and monitoring** | | |
| **2.1 Monitoring of behaviour by others without feedback**  Observe or record behaviour with the person’s knowledge as part of a behaviour change strategy  *Note: if monitoring is part of a data collection procedure rather than a strategy aimed at changing behaviour, do not code; if feedback given, code only* ***2.2, Feedback on behaviour****, and not* ***2.1, Monitoring of behaviour by others without feedback****; if monitoring outcome(s) code* ***2.5, Monitoring outcome(s) of behaviour by others without feedback****; if self-monitoring behaviour, code* ***2.3, Self-monitoring of behaviour*** | Watch hand washing behaviours among health care staff and make notes on context, frequency and technique used | Ask a household member monitor how much the client smokes each day  Ask a pharmacist record if and how often smoking cessation medication is being picked up by the client |
| **2.2 Feedback on behaviour**  Monitor and provide informative or evaluative feedback on performance of the behaviour *(e.g. form, frequency, duration, intensity)*  *Note: if Biofeedback, code only* ***2.6, Biofeedback*** *and not* ***2.2, Feedback on behaviour****; if feedback is on* ***outcome(s)*** *of behaviour, code* ***2.7, Feedback on outcome(s) of behaviour****; if there is no clear evidence that feedback was given, code* ***2.1, Monitoring of behaviour by others without feedback****; if feedback on behaviour is evaluative e.g. praise, also code* ***10.4, Social reward*** | Inform the person of how many steps they walked each day (as recorded on a pedometer) or how many calories they ate each day (based on a food consumption questionnaire). | Give evaluative feedback on the number of cigarettes that the client smoked this week as measured in a daily diary  Give feedback indicating how well the client is adhering to stop smoking medication as reported in a daily diary |
| **2.3 Self-monitoring of behaviour**  Establish a method for the person to monitor and record their behaviour(s) as part of a behaviour change strategy *Note: if monitoring is part of a data collection procedure rather than a strategy aimed at changing behaviour, do not code; if monitoring of outcome of behaviour, code* ***2.4, Self-monitoring of outcome(s) of behaviour****;* *if monitoring is by someone else (without feedback), code* ***2.1, Monitoring of behaviour by others without feedback*** | Ask the person to record daily, in a diary, whether they have brushed their teeth for at least two minutes before going to bed  Give patient a pedometer and a form for recording daily total number of steps | Ask the client to record how many cigarettes they smoke each day in a diary  Ask the client to record daily medication adherence in a diary |
| **2.4 Self-monitoring of outcomes of behaviour**  Establish a method for the person to monitor and record the **outcome(s)** of their behaviour as part of a behaviour change strategy  *Note: if monitoring is part of a data collection procedure rather than a strategy aimed at changing behaviour, do not code ; if monitoring behaviour, code* ***2.3, Self-monitoring of behaviour****; if monitoring is by someone else (without feedback), code* ***2.5, Monitoring outcome(s) of behaviour by others without feedback*** | Ask the person to weigh themselves at the end of each day, over a two-week period, and record their daily weight on a graph to increase exercise behaviours | Ask the client to write down each week of the smoking program how much money they have saved by not smoking |
| **2.5 Monitoring of behaviour by others without feedback**  Observe or record outcomes of behaviour with the person’s knowledge as part of a behaviour change strategy  *Note: if monitoring is part of a data collection procedure rather than a strategy aimed at changing behaviour, do not code; if feedback given, code only* ***2.7, Feedback on outcome(s) of behaviour****; if monitoring behaviour code* ***2.1, Monitoring of behaviour by others without feedback****; if self-monitoring outcome(s), code* ***2.4, Self-monitoring of outcome(s) of behaviour*** | Record blood pressure, blood glucose, weight loss, or physical fitness | Record expired-air carbon monoxide concentration to assess the extent of smoking exposure without giving feedback |
| **2.6 Biofeedback**  Provide feedback about the body *(e.g. physiological or biochemical state)* using an external monitoring device as part of a behaviour change strategy  *Note: if Biofeedback, code only* ***2.6, Biofeedback*** *and not* ***2.2, Feedback on behaviour*** *or* ***2.7, Feedback on outcome(s) of behaviour*** | Inform the person of their blood pressure reading to improve adoption of health behaviours | Inform the person of their measured expired-air carbon monoxide concentration to encourage smoking cessation |
| **2.7 Feedback on outcome(s) of behaviour**  Monitor and provide feedback on the outcome of performance of the behaviour  *Note: if Biofeedback, code only* ***2.6, Biofeedback*** *and not* ***2.7, Feedback on outcome(s) of behaviour****; if feedback is on* ***behaviour*** *code* ***2.2, Feedback on behaviour****; if there is no clear evidence that feedback was given code* ***2.5, Monitoring outcome(s) of behaviour by others without feedback;***  *if feedback on behaviour is evaluative e.g. praise, also code* ***10.4, Social reward*** | Inform the person of how much weight they have lost following the implementation of a new exercise regime | Inform the client about the amount of money they have saved by not smoking during the past weeks |
| **3. Social support** | | |
| **3.1 Social support (unspecified)**  Advise on, arrange or provide social support *(e.g. from friends, relatives, colleagues,’ buddies’ or staff)* or non-contingent praise or reward for performance of the behaviour*.* It includes encouragement and counselling, but only when it is directed at the **behaviour**  *Note: attending a group class and/or mention of ‘follow-up’ does not necessarily apply this BCT, support must be explicitly mentioned; if practical, code* ***3.2, Social support (practical)****; if emotional, code* ***3.3, Social support (emotional)*** (includes ‘**Motivational interviewing**’ and **‘Cognitive Behavioural Therapy’**) | Advise the person to call a ‘buddy’ when they experience an urge to smoke  Arrange for a housemate to encourage continuation with the behaviour change programme  Give information about a self-help group that offers support for the behaviour | Advise the client to ask for social support during the quit attempt from family members, friends, or colleagues  Advise the person to call a ‘buddy’ when they experience an urge to smoke |
| **3.2 Social support (practical)**  Advise on, arrange, or provide **practical** help *(e.g. from friends, relatives, colleagues, ‘buddies’ or staff)* for performance of the behaviour  *Note: if emotional, code* ***3.3, Social support (emotional)****; if general or unspecified, code* ***3.1, Social support (unspecified)*** *If only restructuring the physical environment or adding objects to the environment, code* ***12.1, Restructuring the physical environment*** *or* ***12.5, Adding objects to the environment;*** *attending a group or class and/or mention of ‘follow-up’ does not necessarily apply this BCT, support must be explicitly mentioned.* | Ask the partner of the patient to put their tablet on the breakfast tray so that the patient remembers to take it | Advise client to find someone to accompany them to a new non-smoking venue.  Advise client to ask partner to remove all smoking materials from their shared home.  Advise the client to get a friend, relative or colleague to drive them to the pharmacy to get stop smoking medication, if it is difficult to get to the pharmacy.  Ask the partner of the client to put their stop smoking medication on the breakfast tray so that the client remembers to take it. |
| **3.3 Social support (emotional)**  Advise on, arrange, or provide **emotional** social support *(e.g. from friends, relatives, colleagues, ‘buddies’ or staff)* for performance of the behaviour  *Note: if practical, code* ***3.2, Social support (practical)****; if unspecified, code* ***3.1, Social support (unspecified)*** | Ask the patient to take a partner or friend with them to their colonoscopy appointment | Advise the client to contact a ‘buddy’ to provide emotional support if the client has a difficult time staying abstinent. |
| **4. Shaping knowledge** | | |
| **4.1 Instruction how to perform the behaviour**  Advise or agree on how to perform the behaviour (includes ‘**Skills training**’)  *Note: when the person attends classes such as exercise or cookery, code* ***4.1, Instruction on how to perform the behaviour, 8.1, Behavioural practice/rehearsal*** *and* ***6.1, Demonstration of the behaviour*** | Advise the person how to put a condom on a model of a penis correctly | Advise that the most effective way of quitting is to stop abruptly rather than cut down gradually  Advise how to use a Nicotine Replacement Therapy (NTR) product (e.g. an inhaler) |
| **4.2 Information about antecedents**  Provide information about antecedents  (*e.g. social and environmental situations and events, emotions, cognitions)* that reliably predict performance of the behaviour | Advise to keep a record of snacking and of situations or events occurring prior to snacking | Provide information about the situations, events and emotions that have led the client to starting smoking again  Advise the client to keep a record of situations in which they crave a cigarette |
| **4.3 Re-attribution**  Elicit perceived causes of behaviour and suggest alternative explanations *(e.g. external or internal and stable or unstable)* | If the person attributes their over-eating to the frequent presence of delicious food, suggest that the ‘real’ cause may be the person’s inattention to bodily signals of hunger and satiety | If the person attributes their smoking to the need for stress reduction, suggest that the real cause may be nicotine dependence |
| **4.4 Behavioural experiments**  Advise on how to identify and test hypotheses about the behaviour, its causes and consequences, by collecting and interpreting data | Ask a family physician to give evidence-based advice rather than prescribe antibiotics and to note whether the patients are grateful or annoyed | If the client expects urges to smoke will become unbearable if they do not smoke, advise them to test this by waiting until urges dissipate when feeling the urge to smoke. |
| **4.5 Tell to act**  Tell the person to perform the target behaviour. This would normally involve an expectation of compliance, especially if given by someone with formal or informal authority. |  | Tell the client to stop smoking.  Advise client to take stop smoking medication. |
| **5. Natural consequences** | | |
| **5.1 Information about health consequences**  Provide information (e.g. written, verbal, visual) about health consequences of performing the behaviour  *Note: consequences can be for any target, not just the recipient(s) of the intervention; emphasising importance of consequences is not sufficient; if information about emotional consequences, code* ***5.6, Information about emotional consequences****; if about social, environmental or unspecified consequences code* ***5.3,*** ***Information about social and environmental consequences*** | Explain that not finishing a course of antibiotics can increase susceptibility to future infection  Present the likelihood of contracting a sexually transmitted infection following unprotected sexual behaviour | Give the client a leaflet on the health consequences of smoking |
| **5.2 Salience of consequences**  Use methods specifically designed to **emphasise** the consequences of performing the behaviour with the aim of making them more memorable (goes beyond informing about consequences)  *Note: if information about consequences, also code* ***5.1, Information about health consequences****,* ***5.6, Information about emotional consequences*** *or* ***5.3, Information about social and environmental consequences*** | Produce cigarette packets showing pictures of health consequences e.g. diseased lungs, to highlight the dangers of continuing to smoke | Produce cigarette packets showing pictures of health consequences (e.g. diseased lungs), to highlight the dangers of continuing to smoke  Show the client a ‘tar jar’ which contains all tar that passes through the lungs in a year, to increase awareness of the health consequences of smoking |
| **5.3 Information about social and environmental consequences**  Provide information (e.g. written, verbal, visual) about social and environmental consequences of performing the behaviour  *Note: consequences can be for any target, not just the recipient(s) of the intervention; if information about health or consequences, code* ***5.1, Information about health consequences****; if about emotional consequences, code* ***5.6, Information about emotional consequences****; if unspecified, code* ***5.3, Information about social and environmental consequences*** | Tell family physician about financial remuneration for conducting health screening  Inform a smoker that the majority of people disapprove of smoking in public places | Provide information to the client about the negative health consequences of second-hand smoke to other people  Tell the client that cigarette butts are toxic to the natural environment |
| **5.4 Monitoring of emotional consequences**  Prompt assessment of **feelings** after attempts at performing the behaviour | Agree that the person will record how they feel after taking their daily walk | Ask the client to record how they feel each morning after the quit attempt |
| **5.5 Anticipated regret**  Induce or raise awareness of expectations of future regret about performance of the unwanted behaviour  *Note: not including* ***5.6, Information about emotional consequences***; *if suggests adoption of a perspective or new perspective in order to change cognitions also code* ***13.2, Framing/reframing*** | Ask the person to assess the degree of regret they will feel if they do not quit smoking | Ask the client to assess the degree of regret they will feel if they do not quit smoking |
| **5.6 Information about emotional consequences**  Provide information (e.g. written, verbal, visual) about emotional consequences of performing the behaviour  *Note: consequences can be related to emotional health disorders (e.g. depression, anxiety) and/or states of mind (e.g. low mood, stress); not including* ***5.5, Anticipated regret****; consequences can be for any target, not just the recipient(s) of the intervention; if information about health consequences code* ***5.1, Information about health consequences****; if about social, environmental or unspecified code* ***5.3, Information about social and environmental consequences*** | Explain that quitting smoking increases happiness and life satisfaction | Explain that quitting smoking increases happiness and life-satisfaction |
| **6. Comparison of behaviour** | | |
| **6.1 Demonstration of the behaviour**  Provide an observable sample of the performance of the behaviour, directly in person or indirectly e.g. via film, pictures, for the person to aspire to or imitate (includes ‘**Modelling**’). *Note:* if advised to practice, also code, ***8.1, Behavioural practice and rehearsal;*** *If provided with instructions on how to perform, also code* ***4.1, Instruction on how to perform the behaviour*** | Demonstrate to nurses how to raise the issue of excessive drinking with patients via a role-play exercise | Identify examples of celebrities who have quit smoking.  Demonstrate to the client how to use a specific Nicotine Replacement Therapy product (e.g. an inhaler) |
| **6.2 Social comparison**  Draw attention to others’ performance to allow comparison with the person’s own performance *Note:* *being in a group setting does not necessarily mean that social comparison is actually taking place* | Show the doctor the proportion of patients who were prescribed antibiotics for a common cold by other doctors and compare with their own data | Show the proportion of patients who have had previous failed quit attempts and have successfully quit smoking  Draw attention to how well other group members have managed to use stop smoking medication and compare this with the person’s own performance |
| **6.3 Information about others’ approval**  Provide information about what other people think about the behaviour. The information clarifies whether others will like, approve or disapprove of what the person is doing or will do. | Tell the staff at the hospital ward that staff at all other wards approve of washing their hands according to the guidelines | Tell the client that most people disapprove of smoking in public places  Explain that children are usually happy to hear that their parent is quitting smoking |
| **7. Associations** | | |
| **7.1 Prompts/cues**  Introduce or define environmental or social stimulus with the purpose of prompting or cueing the behaviour. The prompt or cue would normally occur at the time or place of performance  *Note: when a stimulus is linked to a specific action in an if-then plan including one or more of frequency, duration or intensity* *also code* ***1.4, Action planning****.* | Put a sticker on the bathroom mirror to remind people to brush their teeth | Place some alternative to cigarettes e.g. sweets, where cigarettes would normally have been kept.  Send a text message every morning to remind the client to take stop smoking medication  Advise the client to put stop smoking medication next to a toothbrush to remind them to take it in the morning |
| **7.2 Cue signalling reward**  Identify an environmental stimulus that reliably predicts that reward will follow the behaviour (includes ***‘*Discriminative cue’**) | Advise that a fee will be paid to dentists for a particular dental treatment of 6-8-year-old, but not older, children to encourage delivery of that treatment (the 6-8-year-old children are the environmental stimulus) | Advise that a financial incentive will be paid if the client is abstinent from smoking until delivery of the baby but not earlier |
| **7.3 Reduce prompts/cues**  Withdraw gradually prompts to perform the behaviour (includes ***‘*Fading*’***) | Reduce gradually the number of reminders used to take medication | Gradually reduce the number of days on which the client records data such as craving or goal progression which have been used to prompt smoking cessation.  Gradually reduce the number of reminders (e.g. text messages) the client receives to take their stop smoking medication |
| **7.4 Remove access to the reward**  Advise or arrange for the person to be separated from situations in which unwanted behaviour can be rewarded in order to reduce the behaviour (includes ***‘*Time out’**) | Arrange for cupboard containing high calorie snacks to be locked for a specified period to reduce the consumption of sugary foods in between meals | Advise client to remove all cigarettes from the house |
| **7.5 Remove aversive stimulus**  Advise or arrange for the removal of an aversive stimulus to facilitate behaviour change (includes ***‘*Escape learning*’***) | Arrange for a gym-buddy to stop nagging the person to do more exercise in order to increase the desired exercise behaviour | Arrange for the removal of something the client finds unpleasant (e.g. household chores) on adhering to stop smoking medication for a specified period of time |
| **7.6 Satiation**  Advise or arrange repeated exposure to a stimulus that reduces or extinguishes a drive for the unwanted behaviour | Arrange for the person to eat large quantities of chocolate, in order to reduce the person’s appetite for sweet foods | Advise a person to smoke much more than usual, to extinguish the urge to smoke |
| **7.7 Exposure**  Provide systematic confrontation with a feared stimulus to reduce the response to a later encounter | Agree a schedule by which the person who is frightened of surgery will visit the hospital where they are scheduled to have surgery | Agree a schedule by which a client, who fears they will start smoking again when visiting a pub, will systematically visit pubs to reduce this fear. |
| **7.8 Associative learning**  Present a neutral stimulus jointly with a stimulus that already elicits the behaviour repeatedly until the neutral stimulus elicits that behaviour (includes ***‘*Classical/Pavlovian Conditioning’**)  *Note: when a BCT involves reward or punishment, code one or more of:* ***10.2, Material reward (behaviour); 10.3, Non-specific reward; 10.4, Social reward, 10.9, Self-reward; 10.10, Reward (outcome)*** | Present repeatedly fatty foods with a disliked sauce to discourage the consumption of fatty foods | When the client does not smoke when feeling sick; ask them to smoke rapidly, until they feel nauseous, so that smoking becomes associated with feeling sick. |
| **8. Repetition and substitution** | | |
| **8.1 Behavioural practice/rehearsal**  Prompt practice or rehearsal of the performance of the behaviour one or more times in a context or at a time when the performance may not be necessary, in order to increase habit and skill  *Note: if aiming to associate performance with the context, also code* ***8.3, Habit formation*** | Prompt asthma patients to practice measuring their peak flow in the nurse’s consulting room | Advise to practice or rehearse not smoking in a context or time prior to the quit date, to increase the habit of not smoking |
| **8.2 Behaviour substitution**  Prompt substitution of the unwanted behaviour with a wanted or neutral behaviour  *Note: if this occurs regularly, also code* ***8.4, Habit reversal*** | Suggest that the person goes for a walk rather than watches television | Prompt the client to substitute smoking a cigarette with a wanted or neutral behaviour (e.g. brief physical exercise, eating a lollipop) |
| **8.3 Habit formation**  Prompt rehearsal and repetition of the behaviour in the same context repeatedly so that the context elicits the behaviour  *Note: also code* ***8.1, Behavioural practice/rehearsal*** | Prompt patients to take their statin tablet before brushing their teeth every evening | Advise to practice or rehearse not smoking in the same context repeatedly, so that the context elicits abstinence |
| **8.4 Habit reversal**  Prompt rehearsal and repetition of an alternative behaviour to **replace** an unwanted habitual behaviour  *Note: also code* ***8.2, Behaviour substitution*** | Ask the person to walk up stairs at work where they previously always took the lift | Prompt repetition of a wanted or neutral behaviour (e.g. brief physical exercise, eating a lollipop) at the time or places where the client would previously have smoked cigarettes |
| **8.5 Generalisation of target behaviour**  Advise to perform the wanted behaviour, which is already performed in a particular situation, in another situation | Advise to repeat toning exercises learned in the gym when at home | Advise that since people already do not smoke in certain situations (e.g., on during travel in public transport, during dinner) to extend these non-smoking situations to another situation in which they usually smoke |
| **8.6 Graded tasks**  Set easy-to-perform tasks, making them increasingly difficult, but achievable, until behaviour is performed | Ask the person to walk for 100 yards a day for the first week, then half a mile a day after they have successfully achieved 100 yards, then two miles a day after they have successfully achieved one mile | Ask the client to get through a morning without smoking, then to get through a whole day without smoking until one has achieved a larger target of not smoking for one week |
| **9. Comparison of outcomes** | | |
| **9.1 Credible source**  Present verbal or visual communication from a credible source **in favour of or against the behaviour**  *Note:* *code this BCT if source generally agreed on as credible e.g., health professionals, celebrities or words used to indicate expertise or leader in field and if the communication has the aim of persuading;*  *if information about health consequences, also code* ***5.1, Information about health consequences****, if about emotional consequences, also code* ***5.6, Information about emotional consequences****; if about social, environmental or unspecified consequences also code* ***5.3, Information about social and environmental consequences*** | Present a speech given by a high-status professional to emphasise the importance of not exposing patients to unnecessary radiation by ordering x-rays for back pain | Present verbal or visual communication from a high-status health expert in favour of smoking cessation |
| **9.2 Pros and cons**  Advise the person to identify and compare reasons for wanting (pros) and not wanting to (cons) change the behaviour (includes ‘**Decisional balance’***)*  *Note:* *if providing information about health consequences, also code* ***5.1, Information about health consequences****; if providing information about emotional consequences, also code* ***5.6, Information about emotional consequences****; if providing information about social, environmental or unspecified consequences also code* ***5.3, Information about social and environmental consequences*** | Advise the person to list and compare the advantages and disadvantages of prescribing antibiotics for upper respiratory tract infections | Advise the client to generate a list of reasons why they do and why they do not want to stop smoking |
| **9.3 Comparative imagining of future outcomes**  Prompt or advise the imagining and comparing of future outcomes of changed versus unchanged behaviour | Prompt the person to imagine and compare likely or possible outcomes following attending versus not attending a screening appointment | Prompt the client to imagine the possible future in which they have stopped smoking and compare it with a future where they continue to smoke. |
| **10. Reward and threat** | | |
| **10.1 Material incentive (behaviour)**  Inform that money, vouchers or other valued objects ***will be*** delivered if and only if there has been effort and/or progress in performing the behaviour (includes ***‘*Positive reinforcement’**)  *Note: if incentive is social, code* ***10.5, Social incentive*** *if unspecified code* ***10.6,*** ***Non-specific incentive,*** *and not* ***10.1, Material incentive (behaviour)****; if incentive is for* ***outcome,*** *code* ***10.8, Incentive (outcome).*** *If reward is delivered also code one of:* ***10.2, Material reward (behaviour); 10.3, Non-specific reward; 10.4, Social reward, 10.9, Self-reward; 10.10, Reward (outcome)*** | Inform that a financial payment will be made each month in pregnancy that the woman has not smoked | Inform that a financial payment will be made each month in pregnancy that the woman has not smoked  Inform the client that vouchers will be given for each week in which stop smoking medication is used correctly |
| **10.2 Material reward (behaviour)**  Arrange for the delivery of money, vouchers or other valued objects if and only if there ***has been*** effort and/or progress in performing the behaviour (includes ‘**Positive reinforcement’**)  *Note: If reward is social, code* ***10.4, Social reward****, if unspecified code* ***10.3, Non-specific reward****, and not* ***10.1, Material reward (behaviour)****; if reward is for* ***outcome****, code* ***10.10, Reward (outcome).*** *If informed of reward in advance of rewarded behaviour, also code one of:* ***10.1, Material incentive (behaviour); 10.5, Social incentive; 10.6, Non-specific incentive; 10.7, Self-incentive; 10.8, Incentive (outcome)*** | Arrange for the person to receive money that would have been spent on cigarettes if and only if the smoker has not smoked for one month | Arrange for the person to receive money that would have been spent on cigarettes if and only if the smoker has not smoked for one month |
| **10.3 Non-specific reward**  Arrange delivery of a reward if and only if there ***has been*** effort and/or progress in performing the behaviour (includes ‘**Positive reinforcement’**)  *Note: if reward is material, code* ***10.2, Material reward (behaviour)****, if social, code* ***10.4, Social reward****, and not* ***10.3, Non-specific reward****; if reward is for* ***outcome*** *code* ***10.10, Reward (outcome).*** *If informed of reward in advance of rewarded behaviour, also code one of****: 10.1, Material incentive (behaviour); 10.5, Social incentive; 10.6, Non-specific incentive; 10.7, Self-incentive; 10.8, Incentive (outcome)*** | Identify something (e.g. an activity such as a visit to the cinema) that the person values and arrange for this to be delivered if and only if they attend for health screening | Identify something (e.g. an activity such as a visit to the cinema) that the person values and arrange for this to be delivered if and only if they have stayed abstinent for a week |
| **10.4 Social reward (behaviour)**  Arrange verbal or non-verbal reward if and only if there ***has been*** effort and/or progress in performing the behaviour (includes ‘**Positive reinforcement**’)  *Note: if reward is material, code* ***10.2, Material reward (behaviour)****, if unspecified code* ***10.3, Non-specific reward****, and not* ***10.4, Social reward****; if reward is for* ***outcome*** *code* ***10.10, Reward (outcome).*** *If informed of reward in advance of rewarded behaviour, also code one of****: 10.1, Material incentive (behaviour); 10.5, Social incentive; 10.6, Non-specific incentive; 10.7, Self-incentive; 10.8, Incentive (outcome)*** | Congratulate the person for each day they eat a reduced fat diet | Congratulate the person for each week they do not smoke  Give praise or encouragement for improving their adherence to stop smoking medication |
| **10.5 Social incentive**  Inform that a verbal or non-verbal reward ***will be*** delivered if and only if there has been effort and/or progress in performing the behaviour (includes ‘**Positive reinforcement’**)  *Note: if incentive is material, code* ***10.1, Material incentive (behaviour)****, if unspecified code* ***10.6, Non-specific incentive****, and not* ***10.5, Social incentive****; if incentive is for* ***outcome*** *code* ***10.8, Incentive (outcome).*** *If reward is delivered also code one of****: 10.2, Material reward (behaviour); 10.3, Non-specific reward; 10.4, Social reward, 10.9, Self-reward; 10.10, Reward (outcome)*** | Inform that they will be congratulated for each day they eat a reduced fat diet | Inform the client that they will be congratulated by their partner each week they do not smoke |
| **10.6 Non-specific incentive**  Inform that a reward ***will be*** delivered if and only if there has been effort and/or progress in performing the behaviour (includes ‘**Positive reinforcement’**)  *Note: if incentive is material, code* ***10.1, Material incentive (behaviour)****, if social, code* ***10.5, Social incentive*** *and not* ***10.6, Non-specific incentive****; if incentive is for* ***outcome*** *code* ***10.8, Incentive (outcome).*** *If reward is delivered also code one of****: 10.2, Material reward (behaviour); 10.3, Non-specific reward; 10.4, Social reward, 10.9, Self-reward; 10.10, Reward (outcome)*** | Identify an activity that the person values and inform them that this will happen if and only if they attend for health screening | Inform the client that something that they value (not money, e.g. an activity such as a visit to the cinema) will be given if and only if they have not smoked for a month |
| **10.7 Self-incentive**  Plan to reward self in future if and only if there has been effort and/or progress in performing the behaviour  *Note: if self-reward is material, also code* ***10.1, Material incentive (behaviour)****, if social, also code* ***10.5, Social incentive****, if unspecified, also code* ***10.6, Non-specific incentive****; if incentive is for* ***outcome*** *code* ***10.8, Incentive (outcome).*** *If reward is delivered also code one of:* ***10.2, Material reward (behaviour); 10.3, Non-specific reward; 10.4, Social reward, 10.9, Self-reward; 10.10, Reward (outcome)*** | Encourage to provide self with material (e.g., new clothes) or other valued objects if and only if they have adhered to a healthy diet | Ask the client to make a plan to give them self a reward (e.g. nice clothes) if and only if they have not smoked for 2 weeks |
| **10.8 Incentive (outcome)**  Inform that a reward ***will be*** delivered if and only if there has been effort and/or progress in achieving the behavioural **outcome** (*includes* ***‘*Positive reinforcement*’***)  *Note: this includes social, material, self- and non-specific incentives for outcome; if incentive is for the* ***behaviour*** *code* ***10.5****,* ***Social*** ***incentive****,* ***10.1, Material*** ***incentive (behaviour)****,* ***10.6, Non****-****specific incentive*** *or* ***10.7****,* ***Self****-****incentive*** *and not* ***10.8, Incentive (outcome).*** *If reward is delivered also code one of:* ***10.2, Material reward (behaviour); 10.3, Non-specific reward; 10.4, Social reward, 10.9, Self-reward; 10.10, Reward (outcome)*** | Inform the person that they will receive money if and only if a certain amount of weight is lost | Inform the client that a reward will be delivered if and only if they have made progress in reducing CO-levels of expired air. |
| **10.9 Self-reward**  Prompt self-praise or self-reward if and only if there ***has been*** effort and/or progress in performing the behaviour  *Note: if self-reward is material, also code* ***10.2, Material reward (behaviour)****, if social, also code* ***10.4, Social reward****, if unspecified, also code* ***10.3, Non-specific reward****; if reward is for* ***outcome*** *code* ***10.10, Reward (outcome).*** *If informed of reward in advance of rewarded behaviour, also code one of:* ***10.1, Material incentive (behaviour); 10.5, Social incentive; 10.6, Non-specific incentive; 10.7, Self-incentive; 10.8, Incentive (outcome)*** | Encourage to reward self with material (e.g., new clothes) or other valued objects if and only if they have adhered to a healthy diet | Encourage the client to praise themselves if and only if they have been abstinent for 2 days |
| **10.10 Reward (outcome)**  Arrange for the delivery of a reward if and only if there ***has been*** effort and/or progress in achieving the behavioural **outcome** (includes ‘**Positive reinforcement**’)  *Note: this includes social, material, self- and non-specific rewards for outcome; if reward is for the* ***behaviour*** *code* ***10.4****,* ***Social*** ***reward****,* ***10.2, Material*** ***reward (behaviour)****,* ***10.3,*** ***Non****-****specific*** ***reward*** *or* ***10.9****,* ***Self****-****reward*** *and not* ***10.10, Reward (outcome).*** *If informed of reward in advance of rewarded behaviour, also code one of****: 10.1, Material incentive (behaviour); 10.5, Social incentive; 10.6, Non-specific incentive; 10.7, Self-incentive; 10.8, Incentive (outcome)*** | Arrange for the person to receive money if and only if a certain amount of weight is lost | Arrange for the client to save all the money they would have spent on cigarettes and buy something they value with it (e.g. a book) if and only if they have received low readings on a carbon monoxide reader for a month |
| **10.11 Future punishment**  Inform that future punishment or removal of reward will be a consequence of performance of an unwanted behaviour (may include fear arousal) (includes ***‘*Threat*’***) | Inform that continuing to consume 30 units of alcohol per day is likely to result in loss of employment if the person continues | Inform the client that smoking at work is likely to result in loss of employment with the NHS |
| **11. Regulation** | | |
| **11.1 Pharmacological support**  Provide, or encourage the use of or adherence to, drugs to facilitate behaviour change  *Note: if pharmacological support to reduce negative emotions (i.e. anxiety) then also code* ***11.2, Reduce negative emotions*** | Suggest the patient asks the family physician for nicotine replacement therapy to facilitate smoking cessation | Enact the necessary procedures to ensure that the client gets their stop smoking medication easily and without charge where appropriate |
| **11.2 Reduce negative emotions**  Advise on ways of reducing negative emotions to facilitate performance of the behaviour (includes ‘**Stress Management**’)  *Note: if includes analysing the behavioural problem, also code* ***1.2****,* ***Problem solving*** | Advise on the use of stress management skills, e.g. to reduce anxiety about joining Alcoholics Anonymous | Advise on the use of specific relaxation techniques to the client to make it easier to stay abstinent |
| **11.3 Conserving mental resources**  Advise on ways of minimising demands on mental resources to facilitate behaviour change | Advise to carry food calorie content information to reduce the burden on memory in making food choices | Advise the client to relax as much as possible and get a good night’s sleep while trying to stop smoking. |
| **11.4 Paradoxical instructions**  Advise to engage in some form of the unwanted behaviour with the aim of reducing motivation to engage in that behaviour | Advise a smoker to smoke twice as many cigarettes a day as they usually do  Tell the person to stay awake as long as possible in order to reduce insomnia | Advise the client to smoke twice as many cigarettes as they usually do to reduce the motivation to smoke |
| **12. Antecedents** | | |
| **12.1 Restructuring the physical environment**  Change, or advise to change the **physical** environment in order to facilitate performance of the wanted behaviour or create barriers to the unwanted behaviour (other than prompts/cues, rewards and punishments)  *Note: this may also involve* ***12.3, Avoidance/reducing exposure to cues for the behaviour****;* *if restructuring of the social environment code* ***12.2, Restructuring the social environment;***  *if only adding objects to the environment, code* ***12.5, Adding objects to the environment*** | Advise to keep biscuits and snacks in a cupboard that is inconvenient to get to  Arrange to move vending machine out of the school | Advise the client to remove all tobacco products, lighters and ashtrays from their surroundings |
| **12.2 Restructuring the social environment**  Change, or advise to change the **social** environment in order to facilitate performance of the wanted behaviour or create barriers to the unwanted behaviour (other than prompts/cues, rewards and punishments)  *Note: this may also involve* ***12.3, Avoidance/reducing exposure to cues for the behaviour****; if also restructuring of the physical environment also code* ***12.1, Restructuring the physical environment*** | Advise to minimise time spent with friends who drink heavily to reduce alcohol consumption | Advise the client to spend less time with friends who smoke.  Advise the client to ask smoking family members, housemates, friends and/or colleagues not to smoke in their presence |
| **12.3 Avoidance/reducing exposure to cues for the behaviour**  Advise on how to avoid exposure to specific social and contextual/physical cues for the behaviour, including changing daily or weekly routines  *Note:* *this may also involve* ***12.1, Restructuring the physical environment*** and/or ***12.2, Restructuring the social environment***; if the BCT includes analysing the behavioural problem, only code ***1.2*,** ***Problem solving*** | Suggest to a person who wants to quit smoking that their social life focus on activities other than pubs and bars which have been associated with smoking | Advise the client to avoid situations such as pubs in which common triggers to smoke occur by changing daily/weekly routines |
| **12.4 Distraction**  Advise or arrange to use an alternative focus for attention to avoid triggers for the unwanted behaviour | Suggest to a person who is trying to avoid between-meal snacking to focus on a topic they enjoy (e.g. holiday plans) instead of focusing on food | Suggest to a person who is trying to quit smoking to focus on a topic they enjoy (e.g. holiday plans) when they feel the urge to smoke |
| **12.5 Adding objects to the environment**  Add objects to the environment in order to facilitate performance of the behaviour  *Note: Provision of information (e.g. written, verbal, visual) in a booklet or leaflet is insufficient. If this is accompanied by social support, also code* ***3.2, Social support (practical)****; if the environment is changed beyond the addition of objects, also code* ***12.1, Restructuring the physical environment*** | Provide free condoms to facilitate safe sex  Provide attractive toothbrush to improve tooth brushing technique | Arrange for cigarette packets to carry anti-smoking messages and images to encourage reduced smoking rates  Give the client an electronic medication monitoring device, which indicates whether or not they have taken their medication today. |
| **13. Identity** | | |
| **13.1 Identification of self as a role-model**  Inform that one's own behaviour may be an example to others | Inform the person that if they eat healthily, that may be a good example for their children | Inform the client that if they quit smoking, they would be a good role-model for smoking friends |
| **13.2 Framing/reframing**  Suggest the deliberate adoption of a perspective or new perspective on behaviour (e.g. its purpose) in order to change cognitions or emotions about performing the behaviour (includes ‘**Cognitive structuring**’)  *Note: If information about consequences then code* ***5.1, Information about health consequences, 5.6, Information about emotional consequences*** *or* ***5.3, Information about social and environmental consequences*** *instead of* ***13.2, Framing/reframing*** | Suggest that the person might think of the tasks as reducing sedentary behaviour (rather than increasing activity) | Suggest the client deliberately adopts a different, more positive view on past failed quit attempts or lapses (e.g. advise that lapses can be viewed as a valuable learning experience that can help with this quit attempt) |
| **13.3 Incompatible beliefs**  Draw attention to discrepancies between current or past behaviour and self-image, in order to create discomfort (includes ***‘*Cognitive dissonance’**) | Draw attention to a doctor’s liberal use of blood transfusion and their self-identification as a proponent of evidence-based medical practice | Draw attention to the fact that the client sees themselves as athletic, but at the same time is reducing their athletic ability by smoking |
| **13.4 Valued self-identity**  Advise the person to write or complete rating scales about a cherished value or personal strength as a means of affirming the person’s identity as part of a behaviour change strategy (includes ***‘*Self-affirmation’**) | Advise the person to write about their personal strengths before they receive a message advocating the behaviour change | Advise the client to write or talk about a cherished value or personal strength as a means to affirming their identity in order to strengthen their ability to quit smoking |
| ***13.5 Identity associated with changed behaviour***  Advise the person to construct a new self-identity as someone who ‘used to engage with the unwanted behaviour’ | Ask the person to articulate their new identity as an ‘ex-smoker’ | Ask the person to articulate their new identity as an ‘ex-smoker’ |
| **14. Scheduled consequences** | | |
| **14.1 Behaviour cost**  Arrange for withdrawal of something valued if and only if an unwanted behaviour is performed (includes ‘**Response cost’**). Note if withdrawal of contingent reward code*,* ***14.3, Remove reward*** | Subtract money from a prepaid refundable deposit when a cigarette is smoked | Subtract money from a prepaid refundable deposit when a cigarette is smoked |
| **14.2 Punishment**  Arrange for aversive consequence contingent on the performance of the unwanted behaviour | Arrange for the person to wear unattractive clothes following consumption of fatty foods | Arrange that the client has to do their housemates tasks (e.g. the laundry) when they smoke a cigarette this week |
| **14.3 Remove reward**  Arrange for discontinuation of contingent reward following performance of the unwanted behaviour (includes **‘Extinction’**) | Arrange for the other people in the household to ignore the person every time they eat chocolate (rather than attending to them by criticising or persuading) | Arrange for household members to ignore the client if the client is smoking a cigarette |
| **14.4 Reward approximation**  Arrange for reward following any approximation to the target behaviour, gradually rewarding only performance closer to the wanted behaviour (includes ***‘*Shaping*’***) | Arrange reward for any reduction in daily calories, gradually requiring the daily calorie count to become closer to the planned calorie intake | Arrange for money to be given to the client each week they successfully cut down smoking by any number of cigarettes gradually requiring this reduction in number of cigarettes to be greater and approach cessation. |
| **14.5 Rewarding completion**  Build up behaviour by arranging reward following final component of the behaviour; gradually add the components of the behaviour that occur earlier in the behavioural sequence (includes ***‘*Backward chaining’**)  *Note: also code one of* ***10.2, Material reward (behaviour); 10.3, Non-specific reward; 10.4, Social reward, 10.9, Self-reward; 10.10, Reward (outcome)*** | Reward eating a supplied low-calorie meal; then make reward contingent on cooking and eating the meal; then make reward contingent on purchasing, cooking and eating the meal | Reward quitting in pregnant smokers; then make reward contingent on staying abstinent for a week; then make reward contingent on staying abstinent for a month; then make reward contingent on staying abstinent until the baby is born.  Reward taking supplied stop smoking medication; then make reward contingent on prompted purchasing and taking medication; then make reward contingent on self-initiated purchasing of medication and taking medication. |
| **14.6 Situation-specific reward**  Arrange for reward following the behaviour in one situation but not in another (includes ***‘*Discrimination training’**)  *Note:* *also code one of* ***10.2, Material reward (behaviour); 10.3, Non-specific reward; 10.4, Social reward, 10.9, Self-reward; 10.10, Reward (outcome)*** | Arrange reward for eating at mealtimes but not between meals | Arrange for a reward to be given to the client for not smoking after a meal but do not give a reward for not smoking during a meal (when they would normally not smoke). |
| **14.7 Reward incompatible behaviour**  Arrange reward for responding in a manner that is incompatible with a previous response to that situation (includes ***‘*Counter-conditioning’**)  *Note: also code one of* ***10.2, Material reward (behaviour); 10.3, Non-specific reward; 10.4, Social reward, 10.9, Self-reward; 10.10, Reward (outcome)*** | Arrange reward for ordering a soft drink at the bar rather than an alcoholic beverage | Arrange for client to be rewarded for arranging to meet friends, whom they have previously met in a venue where smoking was allowed, in a non-smoking venue |
| **14.8 Reward alternative behaviour**  Arrange reward for performance of an alternative to the unwanted behaviour (includes ***‘*Differential reinforcement*’***)  *Note: also code one of* ***10.2, Material reward (behaviour); 10.3, Non-specific reward; 10.4, Social reward, 10.9, Self-reward; 10.10, Reward (outcome);*** *consider also coding* ***1.2, Problem solving*** | Reward for consumption of low-fat foods but not consumption of high fat foods | Arrange for the client to receive a reward if he eats a lollipop when feeling the urge to smoke rather than smoking a cigarette.  Arrange for the client to receive a reward if they buy something else (e.g. a magazine) at their local cigarette shop, rather than cigarettes. |
| **14.9 Reduce reward frequency**  Arrange for rewards to be made contingent on increasing duration or frequency of the behaviour (includes ***‘*Thinning*’***)  *Note: also code one of* ***10.2, Material reward (behaviour); 10.3, Non-specific reward; 10.4, Social reward, 10.9, Self-reward; 10.10, Reward (outcome)*** | Arrange reward for each day without smoking, then each week, then each month, then every 2 months and so on | Arrange reward for each day without smoking, then for each week, then for each month, then every two months and so on |
| **14.10 Remove punishment**  Arrange for removal of an unpleasant consequence contingent on performance of the wanted behaviour (includes ***‘*Negative reinforcement’**) | Arrange for someone else to do housecleaning only if the person has adhered to the medication regimen for a week | Arrange that a housemate will take up some of the disliked household tasks (e.g. cleaning) if the client does not smoke for a week |
| **15. Self-belief** | | |
| **15.1 Verbal persuasion about capability**  Tell the person that they can successfully perform the wanted behaviour, arguing against self-doubts and asserting that they can and will succeed | Tell the person that they can successfully increase their physical activity, despite their recent heart attack. | Tell the person that they can quit smoking even though previous quit attempts have not been successful |
| **15.2 Mental rehearsal of successful performance**  Advise to practise imagining performing the behaviour successfully in relevant contexts | Advise to imagine eating and enjoying a salad in a work canteen | Advise the client to imagine successfully not smoking in specific situations in which one usually smokes (e.g. after dinner, at a party). |
| **15.3 Focus on past success**  Advise to think about or list previous successes in performing the behaviour (or parts of it) | Advise to describe or list the occasions on which the person had ordered a non-alcoholic drink in a bar | Ask the client to describe times when they have successfully gone without a cigarette in a situation in which they would normally have smoked |
| **15.4 Self-talk**  Prompt positive self-talk (aloud or silently) before and during the behaviour | Prompt the person to tell themselves that a walk will be energising | Prompt the client to tell themselves when they feel urges to smoke that they will be able to get through the day without smoking  Prompt a person to tell themselves that using stop smoking medication will make them feel better because it reduces withdrawal symptoms |
| **16. Covert learning** | | |
| **16.1 Imaginary punishment**  Advise to imagine performing the **unwanted** behaviour in a real-life situation followed by imagining an unpleasant consequence (includes ***‘*Covert sensitisation’**) | Advise to imagine overeating and then vomiting | Advise the client to imagine starting smoking again and feeling unhealthy, unhappy and low on energy |
| **16.2 Imaginary reward**  Advise to imagine performing the **wanted** behaviour in a real-life situation followed by imagining a pleasant consequence (includes ***‘*Covert conditioning’**) | Advise the health professional to imagine giving dietary advice followed by the patient losing weight and no longer being diabetic | Advise the client to imagine feeling healthier, happier and more energetic after successfully having quit smoking |
| **16.3 Vicarious consequences**  Prompt observation of the consequences (including rewards and punishments) for others when they perform the behaviour  *Note:* *if observation of health consequences, also code* ***5.1, Information about health consequences****; if of emotional consequences, also code* ***5.6, Information about emotional consequences****, if of*  *social, environmental or unspecified consequences, also code* ***5.3,*** ***Information about social and environmental consequences*** | Draw attention to the positive comments other staff get when they disinfect their hands regularly | Draw attention to compliments other people get when they quit smoking |

**Supplemental Figure A1.**

Decision Tree used to Rate the Quality of Comparator (and Experimental) Group Intervention Descriptions

**Yes**

**Yes**

**No**

**No**

**Yes**

**No**

**Supplemental Table A2.**

Preferred Reporting Items for Systematic Reviews and Meta-Analyses (PRISMA) Checklist

| **Section/topic** | **#** | **Checklist item** | **Page** |
| --- | --- | --- | --- |
| **TITLE** | | |  |
| Title | 1 | Identify the report as a systematic review, meta-analysis, or both. | 1 |
| **ABSTRACT** | | |  |
| Structured summary | 2 | Provide a structured summary including, as applicable: background; objectives; data sources; study eligibility criteria, participants, and interventions; study appraisal and synthesis methods; results; limitations; conclusions and implications of key findings; systematic review registration number. | 2 |
| **INTRODUCTION** | | |  |
| Rationale | 3 | Describe the rationale for the review in the context of what is already known. | 3-4 |
| Objectives | 4 | Provide an explicit statement of questions being addressed with reference to participants, interventions, comparisons, outcomes, and study design (PICOS). | 4-5 |
| **METHODS** | | |  |
| Protocol and registration | 5 | Indicate if a review protocol exists, if and where it can be accessed (e.g., Web address), and, if available, provide registration information including registration number. | 4-6 |
| Eligibility criteria | 6 | Specify study characteristics (e.g., PICOS, length of follow-up) and report characteristics (e.g., years considered, language, publication status) used as criteria for eligibility, giving rationale. | 5 |
| Information sources | 7 | Describe all information sources (e.g., databases with dates of coverage, contact with study authors to identify additional studies) in the search and date last searched. | 5 |
| Search | 8 | Present full electronic search strategy for at least one database, including any limits used, such that it could be repeated. | NA |
| Study selection | 9 | State the process for selecting studies (i.e., screening, eligibility, included in systematic review, and, if applicable, included in the meta-analysis). | 9, Protocol |
| Data collection process | 10 | Describe method of data extraction from reports (e.g., piloted forms, independently, in duplicate) and any processes for obtaining and confirming data from investigators. | 5-7 |
| Data items | 11 | List and define all variables for which data were sought (e.g., PICOS, funding sources) and any assumptions and simplifications made. | 5-7 |
| Risk of bias in individual studies | 12 | Describe methods used for assessing risk of bias of individual studies (including specification of whether this was done at the study or outcome level), and how this information is to be used in any data synthesis. | NA |
| Summary measures | 13 | State the principal summary measures (e.g., risk ratio, difference in means). | 7 |
| Synthesis of results | 14 | Describe the methods of handling data and combining results of studies, if done, including measures of consistency (e.g., I^2^) for each meta-analysis. | 7-9 |
| Risk of bias across studies | 15 | Specify any assessment of risk of bias that may affect the cumulative evidence (e.g., publication bias, selective reporting within studies). | NA |
| Additional analyses | 16 | Describe methods of additional analyses (e.g., sensitivity or subgroup analyses, meta-regression), if done, indicating which were pre-specified. | 7-9 |
| **RESULTS** | | |  |
| Study selection | 17 | Give numbers of studies screened, assessed for eligibility, and included in the review, with reasons for exclusions at each stage, ideally with a flow diagram. | Figure 1 |
| Study characteristics | 18 | For each study, present characteristics for which data were extracted (e.g., study size, PICOS, follow-up period) and provide the citations. | A29-A36, attached data |
| Risk of bias within studies | 19 | Present data on risk of bias of each study and, if available, any outcome level assessment (see item 12). | NA |
| Results of individual studies | 20 | For all outcomes considered (benefits or harms), present, for each study: (a) simple summary data for each intervention group (b) effect estimates and confidence intervals, ideally with a forest plot. | Attached data |
| Synthesis of results | 21 | Present results of each meta-analysis done, including confidence intervals and measures of consistency. | NA |
| Risk of bias across studies | 22 | Present results of any assessment of risk of bias across studies (see Item 15). | NA |
| Additional analysis | 23 | Give results of additional analyses, if done (e.g., sensitivity or subgroup analyses, meta-regression [see Item 16]). | 9-12, A37-A42 |
| **DISCUSSION** | | |  |
| Summary of evidence | 24 | Summarize the main findings including the strength of evidence for each main outcome; consider their relevance to key groups (e.g., healthcare providers, users, and policy makers). | 12-15 |
| Limitations | 25 | Discuss limitations at study and outcome level (e.g., risk of bias), and at review-level (e.g., incomplete retrieval of identified research, reporting bias). | 14 |
| Conclusions | 26 | Provide a general interpretation of the results in the context of other evidence, and implications for future research. | 12-16 |
| **FUNDING** | | |  |
| Funding | 27 | Describe sources of funding for the systematic review and other support (e.g., supply of data); role of funders for the systematic review. | 1 |

Appendix B: Additional results information

Studies Included in this Systematic Review

1. Abrantes AM, Bloom EL, Strong DR, et al. A preliminary randomized controlled trial of a behavioral exercise intervention for smoking cessation. *Nicotine Tob Res.* 2014;16(8):1094-1103.

2. Abroms LC, Boal AL, Simmens SJ, Mendel JA, Windsor RA. A randomized trial of Text2Quit: A text messaging program for smoking cessation. *Am J Prev Med.* 2014;47(3):242-250.

3. Abroms LC, Windsor R, Simons-Morton B. Getting young adults to quit smoking: A formative evaluation of the X-Pack Program. *Nicotine Tob Res.* 2008;10(1):27-33.

4. Ahluwalia JS, Okuyemi K, Nollen N, et al. The effects of nicotine gum and counseling among African American light smokers: A 2× 2 factorial design. *Addiction.* 2006;101(6):883-891.

5. Alessi SM, Petry NM. Smoking reductions and increased self-efficacy in a randomized controlled trial of smoking abstinence–contingent incentives in residential substance abuse treatment patients. *Nicotine Tob Res.* 2014;16(11):1436-1445.

6. Allen Jr B, Pederson LL, Leonard EH. Effectiveness of physicians-in-training counseling for smoking cessation in African Americans. *J Natl Med Assoc.* 1998;90(10):597-604.

7. Ames SC, Patten CA, Werch CE, et al. Expressive writing as a smoking cessation treatment adjunct for young adult smokers. *Nicotine Tob Res.* 2007;9(2):185-194.

8. Ames SC, Pokorny SB, Schroeder DR, Tan W, Werch CE. Integrated smoking cessation and binge drinking intervention for young adults: A pilot efficacy trial. *Addict Behav.* 2014;39(5):848-853.

9. An LC, Klatt C, Perry CL, et al. The RealU online cessation intervention for college smokers: A randomized controlled trial. *Prev Med.* 2008;47(2):194-199.

10. Aveyard P, Brown K, Saunders C, et al. Weekly versus basic smoking cessation support in primary care: A randomised controlled trial. *Thorax.* 2007;62(10):898-903.

11. Baker A, Richmond R, Haile M, et al. A randomized controlled trial of a smoking cessation intervention among people with a psychotic disorder. *Am J Psychiatry.* 2006;163(11):1934-1942.

12. Bakkevig O, Steine S, Hafenbrädl Kv, Lærum E. Smoking cessation: A comparative, randomised study between management in general practice and the behavioural programme SmokEnders. *Scand J Prim Health Care.* 2000;18(4):247-251.

13. Becoña E, Vázquez FL. Effectiveness of personalized written feedback through a mail intervention for smoking cessation: A randomized-controlled trial in Spanish smokers. *J Consult Clin Psychol.* 2001;69(1):33-40.

14. Bize R, Willi C, Chiolero A, et al. Participation in a population-based physical activity programme as an aid for smoking cessation: A randomised trial. *Tob Control.* 2010;19(6):488-494.

15. Blebil AQ, Sulaiman SAS, Hassali MA, Dujaili JA, Zin AM. Impact of additional counselling sessions through phone calls on smoking cessation outcomes among smokers in Penang State, Malaysia. *BMC Public Health.* 2014;14(1):460-468.

16. Bobo JK, Mcilvain HE, Lando HA, Walker RD, Leed‐Kelly A. Effect of smoking cessation counseling on recovery from alcoholism: Findings from a randomized community intervention trial. *Addiction.* 1998;93(6):877-887.

17. Bock BC, Fava JL, Gaskins R, et al. Yoga as a complementary treatment for smoking cessation in women. *J Womens Health.* 2012;21(2):240-248.

18. Bock BC, Papandonatos GD, De Dios MA, et al. Tobacco cessation among low-income smokers: Motivational enhancement and nicotine patch treatment. *Nicotine Tob Res.* 2014;16(4):413-422.

19. Borrelli B, Novak S, Hecht J, Emmons K, Papandonatos G, Abrams D. Home health care nurses as a new channel for smoking cessation treatment: Outcomes from project CARES (Community-nurse Assisted Research and Education on Smoking). *Prev Med.* 2005;41(5-6):815-821.

20. Brown J, Michie S, Geraghty AW, et al. Internet-based intervention for smoking cessation (StopAdvisor) in people with low and high socioeconomic status: A randomised controlled trial. *The Lancet Respiratory Medicine.* 2014;2(12):997-1006.

21. Brown RA, Kahler CW, Niaura R, et al. Cognitive–behavioral treatment for depression in smoking cessation. *J Consult Clin Psychol.* 2001;69(3):471-480.

22. Brown RA, Niaura R, Lloyd-Richardson EE, et al. Bupropion and cognitive–behavioral treatment for depression in smoking cessation. *Nicotine Tob Res.* 2007;9(7):721-730.

23. Brown RA, Reed KMP, Bloom EL, et al. Development and preliminary randomized controlled trial of a distress tolerance treatment for smokers with a history of early lapse. *Nicotine Tob Res.* 2013;15(12):2005-2015.

24. Brunner Frandsen N, Sørensen M, Hyldahl TK, Henriksen RM, Bak S. Smoking cessation intervention after ischemic stroke or transient ischemic attack. A randomized controlled pilot trial. *Nicotine Tob Res.* 2011;14(4):443-447.

25. Carmody TP, Delucchi K, Duncan CL, et al. Intensive intervention for alcohol-dependent smokers in early recovery: A randomized trial. *Drug Alcohol Depend.* 2012;122(3):186-194.

26. Carmody TP, Duncan C, Simon JA, et al. Hypnosis for smoking cessation: A randomized trial. *Nicotine Tob Res.* 2008;10(5):811-818.

27. Chan SS, Leung DY, Abdullah AS, et al. Smoking-cessation and adherence intervention among Chinese patients with erectile dysfunction. *Am J Prev Med.* 2010;39(3):251-258.

28. Chan SS, Leung DY, Wong DC, Lau CP, Wong VT, Lam TH. A randomized controlled trial of stage‐matched intervention for smoking cessation in cardiac out‐patients. *Addiction.* 2012;107(4):829-837.

29. Chen J, Chen Y, Chen P, Liu Z, Luo H, Cai S. Effectiveness of individual counseling for smoking cessation in smokers with chronic obstructive pulmonary disease and asymptomatic smokers. *Experimental and Therapeutic Medicine.* 2014;7(3):716-720.

30. Chouinard M-C, Robichaud-Ekstrand S. The effectiveness of a nursing inpatient smoking cessation program in individuals with cardiovascular disease. *Nurs Res.* 2005;54(4):243-254.

31. Ciccolo JT, Dunsiger SI, Williams DM, et al. Resistance training as an aid to standard smoking cessation treatment: A pilot study. *Nicotine Tob Res.* 2011;13(8):756-760.

32. Clark MM, Cox LS, Jett JR, et al. Effectiveness of smoking cessation self-help materials in a lung cancer screening population. *Lung Cancer.* 2004;44(1):13-21.

33. Cropsey K, Eldridge G, Weaver M, Villalobos G, Stitzer M, Best A. Smoking cessation intervention for female prisoners: Addressing an urgent public health need. *Am J Public Health.* 2008;98(10):1894-1901.

34. Dallery J, Raiff BR, Grabinski MJ. Internet‐based contingency management to promote smoking cessation: A randomized controlled study. *J Appl Behav Anal.* 2013;46(4):750-764.

35. Danielsson T, Jones K, Rössner S, Westin Å. Open randomised trial of intermittent very low energy diet together with nicotine gum for stopping smoking in women who gained weight in previous attempts to quitCommentary: Results are unlikely to be as good in routine practice. *BMJ.* 1999;319(7208):490-494.

36. Davis JM, Goldberg SB, Anderson MC, Manley AR, Smith SS, Baker TB. Randomized trial on mindfulness training for smokers targeted to a disadvantaged population. *Subst Use Misuse.* 2014;49(5):571-585.

37. Dent LA, Harris KJ, Noonan CW. Randomized trial assessing the effectiveness of a pharmacist-delivered program for smoking cessation. *Ann Pharmacother.* 2009;43(2):194-201.

38. Dickson-Spillmann M, Haug S, Schaub MP. Group hypnosis vs. relaxation for smoking cessation in adults: A cluster-randomised controlled trial. *BMC Public Health.* 2013;13(1):1227-1235.

39. Elkins G, Marcus J, Bates J, Hasan Rajab M, Cook T. Intensive hypnotherapy for smoking cessation: A prospective study. *Int J Clin Exp Hypn.* 2006;54(3):303-315.

40. Ellerbeck EF, Mahnken JD, Cupertino AP, et al. Effect of varying levels of disease management on smoking cessation: A randomized trial. *Ann Intern Med.* 2009;150(7):437-446.

41. Feeney GF, McPherson A, Connor J, McAlister A, Young R, Garrahy P. Randomized controlled trial of two cigarette quit programmes in coronary care patients after acute myocardial infarction. *Intern Med J.* 2001;31(8):470-475.

42. Froelicher ES, Doolan D, Yerger VB, McGruder CO, Malone RE. Combining community participatory research with a randomized clinical trial: The protecting the hood against tobacco (PHAT) smoking cessation study. *Heart & Lung: The Journal of Acute and Critical Care.* 2010;39(1):50-63.

43. Gilbody S, Peckham E, Man M-S, et al. Bespoke smoking cessation for people with severe mental ill health (SCIMITAR): A pilot randomised controlled trial. *The Lancet Psychiatry.* 2015;2(5):395-402.

44. Gritz ER, Danysh HE, Fletcher FE, et al. Long-term outcomes of a cell phone–delivered intervention for smokers living with HIV/AIDS. *Clin Infect Dis.* 2013;57(4):608-615.

45. Gulliver SB, Kamholz BW, Helstrom AW, Morissette SB, Kahler CW. A preliminary evaluation of adjuncts to motivational interviewing for psychiatrically complex smokers. *Journal of Dual Diagnosis.* 2008;4(4):394-413.

46. Hajek P, Taylor TZ, Mills P. Brief intervention during hospital admission to help patients to give up smoking after myocardial infarction and bypass surgery: Randomised controlled trial. *BMJ.* 2002;324(7329):87-89.

47. Hall SM, Humfleet GL, Muñoz RF, Reus VI, Prochaska JJ, Robbins JA. Using extended cognitive behavioral treatment and medication to treat dependent smokers. *Am J Public Health.* 2011;101(12):2349-2356.

48. Hall SM, Humfleet GL, Muñoz RF, Reus VI, Robbins JA, Prochaska JJ. Extended treatment of older cigarette smokers. *Addiction.* 2009;104(6):1043-1052.

49. Hall SM, Humfleet GL, Reus VI, Muñoz RF, Cullen J. Extended nortriptyline and psychological treatment for cigarette smoking. *Am J Psychiatry.* 2004;161(11):2100-2107.

50. Hall SM, Humfleet GL, Reus VI, Munoz RF, Hartz DT, Maude-Griffin R. Psychological intervention and antidepressant treatment in smoking cessation. *Arch Gen Psychiatry.* 2002;59(10):930-936.

51. Hall SM, Reus VI, Munoz RF, et al. Nortriptyline and cognitive-behavioral therapy in the treatment of cigarette smoking. *Arch Gen Psychiatry.* 1998;55(8):683-690.

52. Hall SM, Tsoh JY, Prochaska JJ, et al. Treatment for cigarette smoking among depressed mental health outpatients: A randomized clinical trial. *Am J Public Health.* 2006;96(10):1808-1814.

53. Halpern SD, French B, Small DS, et al. Randomized trial of four financial-incentive programs for smoking cessation. *N Engl J Med.* 2015;372(22):2108-2117.

54. Hanioka T, Ojima M, Tanaka H, Naito M, Hamajima N, Matsuse R. Intensive smoking-cessation intervention in the dental setting. *J Dent Res.* 2010;89(1):66-70.

55. Hasan FM, Zagarins SE, Pischke KM, et al. Hypnotherapy is more effective than nicotine replacement therapy for smoking cessation: Results of a randomized controlled trial. *Complement Ther Med.* 2014;22(1):1-8.

56. Heil SH, Higgins ST, Bernstein IM, et al. Effects of voucher‐based incentives on abstinence from cigarette smoking and fetal growth among pregnant women. *Addiction.* 2008;103(6):1009-1018.

57. Hennrikus D, Joseph AM, Lando HA, et al. Effectiveness of a smoking cessation program for peripheral artery disease patients: A randomized controlled trial. *J Am Coll Cardiol.* 2010;56(25):2105-2112.

58. Hennrikus DJ, Lando HA, McCarty MC, et al. The TEAM project: The effectiveness of smoking cessation intervention with hospital patients. *Prev Med.* 2005;40(3):249-258.

59. Hickman NJ, Delucchi KL, Prochaska JJ. Treating tobacco dependence at the intersection of diversity, poverty, and mental illness: A randomized feasibility and replication trial. *Nicotine Tob Res.* 2015;17(8):1012-1021.

60. Higgins ST, Heil SH, Solomon LJ, et al. A pilot study on voucher-based incentives to promote abstinence from cigarette smoking during pregnancy and postpartum. *Nicotine Tob Res.* 2004;6(6):1015-1020.

61. Higgins ST, Washio Y, Lopez AA, et al. Examining two different schedules of financial incentives for smoking cessation among pregnant women. *Prev Med.* 2014;68:51-57.

62. Hollands GJ, Whitwell SC, Parker RA, et al. Effect of communicating DNA based risk assessments for Crohn’s disease on smoking cessation: Randomised controlled trial. *BMJ.* 2012;345:e4708.

63. Hughes JR, Solomon LJ, Livingston AE, Callas PW, Peters EN. A randomized, controlled trial of NRT-aided gradual vs. abrupt cessation in smokers actively trying to quit. *Drug Alcohol Depend.* 2010;111(1):105-113.

64. Humerfelt S, Eide G, Kvale G, Aaro L, Gulsvik A. Effectiveness of postal smoking cessation advice: A randomized controlled trial in young men with reduced FEV1 and asbestos exposure. *Eur Respir J.* 1998;11(2):284-290.

65. Humfleet GL, Hall SM, Delucchi KL, Dilley JW. A randomized clinical trial of smoking cessation treatments provided in HIV clinical care settings. *Nicotine Tob Res.* 2013;15(8):1436-1445.

66. Jason LA, Salina D, McMahon SD, Hedeker D, Stockton M. A worksite smoking intervention: A 2 year assessment of groups, incentives and self-help. *Health Educ Res.* 1997;12(1):129-138.

67. Kahler CW, Metrik J, LaChance HR, et al. Addressing heavy drinking in smoking cessation treatment: A randomized clinical trial. *J Consult Clin Psychol.* 2008;76(5):852-862.

68. Killen JD, Fortmann SP, Davis L, Varady A. Nicotine patch and self-help video for cigarette smoking cessation. *J Consult Clin Psychol.* 1997;65(4):663-672.

69. Lacasse Y, Lamontagne R, Martin S, Simard S, Arsenault M. Randomized trial of a smoking cessation intervention in hospitalized patients. *Nicotine Tob Res.* 2008;10(7):1215-1221.

70. Lancaster T, Dobbie W, Vos K, Yudkin P, Murphy M, Fowler G. Randomized trial of nurse-assisted strategies for smoking cessation in primary care. *Br J Gen Pract.* 1999;49(440):191-194.

71. Lando H, Rolnick S, Klevan D, Roski J, Cherney L, Lauger G. Telephone support as an adjunct to transdermal nicotine in smoking cessation. *Am J Public Health.* 1997;87(10):1670-1674.

72. Ledgerwood DM, Arfken CL, Petry NM, Alessi SM. Prize contingency management for smoking cessation: A randomized trial. *Drug Alcohol Depend.* 2014;140:208-212.

73. Leischow SJ, Muramoto ML, Cook GN, Merikle EP, Castellini SM, Otte PS. OTC nicotine patch: Effectiveness alone and with brief physician intervention. *Am J Health Behav.* 1999;23(1):61-69.

74. Leischow SJ, Ranger-Moore J, Muramoto ML, Matthews E. Effectiveness of the nicotine inhaler for smoking cessation in an OTC setting. *Am J Health Behav.* 2004;28(4):291-301.

75. Levine MD, Perkins KA, Kalarchian MA, et al. Bupropion and cognitive behavioral therapy for weight-concerned women smokers. *Arch Intern Med.* 2010;170(6):543-550.

76. Lewis SF, Piasecki TM, Fiore MC, Anderson JE, Baker TB. Transdermal nicotine replacement for hospitalized patients: A randomized clinical trial. *Prev Med.* 1998;27(2):296-303.

77. Lou P, Zhu Y, Chen P, et al. Supporting smoking cessation in chronic obstructive pulmonary disease with behavioral intervention: A randomized controlled trial. *BMC Family Practice.* 2013;14(1):91-100.

78. Maguire T, McElnay J, Drummond A. A randomized controlled trial of a smoking cessation intervention based in community pharmacies. *Addiction.* 2001;96(2):325-331.

79. Marcus BH, Albrecht AE, King TK, et al. The efficacy of exercise as an aid for smoking cessation in women: A randomized controlled trial. *Arch Intern Med.* 1999;159(11):1229-1234.

80. Marcus BH, Lewis BA, Hogan J, et al. The efficacy of moderate-intensity exercise as an aid for smoking cessation in women: A randomized controlled trial. *Nicotine Tob Res.* 2005;7(6):871-880.

81. Martin JE, Calfas KJ, Patten CA, et al. Prospective evaluation of three smoking interventions in 205 recovering alcoholics: One-year results of Project SCRAP-Tobacco. *J Consult Clin Psychol.* 1997;65(1):190-194.

82. May S, West R, Hajek P, McEwen A, McRobbie H. Randomized controlled trial of a social support (‘buddy’) intervention for smoking cessation. *Patient Educ Couns.* 2006;64(1):235-241.

83. McBride CM, Scholes D, Grothaus LC, Curry SJ, Ludman E, Albright J. Evaluation of a minimal self-help smoking cessation intervention following cervical cancer screening. *Prev Med.* 1999;29(2):133-138.

84. McCarthy DE, Piasecki TM, Lawrence DL, et al. A randomized controlled clinical trial of bupropion SR and individual smoking cessation counseling. *Nicotine Tob Res.* 2008;10(4):717-729.

85. McFall M, Saxon AJ, Malte CA, et al. Integrating tobacco cessation into mental health care for posttraumatic stress disorder: A randomized controlled trial. *JAMA.* 2010;304(22):2485-2493.

86. McFall M, Saxon AJ, Thompson CE, et al. Improving the rates of quitting smoking for veterans with posttraumatic stress disorder. *Am J Psychiatry.* 2005;162(7):1311-1319.

87. Miller NH, Smith PM, DeBusk RF, Sobel DS, Taylor CB. Smoking cessation in hospitalized patients: Results of a randomized trial. *Arch Intern Med.* 1997;157(4):409-415.

88. Mohiuddin SM, Mooss AN, Hunter CB, Grollmes TL, Cloutier DA, Hilleman DE. Intensive smoking cessation intervention reduces mortality in high-risk smokers with cardiovascular disease. *Chest.* 2007;131(2):446-452.

89. Molyneux A, Lewis S, Leivers U, et al. Clinical trial comparing nicotine replacement therapy (NRT) plus brief counselling, brief counselling alone, and minimal intervention on smoking cessation in hospital inpatients. *Thorax.* 2003;58(6):484-488.

90. Mueller S, Petitjean S, Wiesbeck G. Cognitive behavioral smoking cessation during alcohol detoxification treatment: A randomized, controlled trial. *Drug Alcohol Depend.* 2012;126(3):279-285.

91. Murray RL, Coleman T, Antoniak M, et al. The effect of proactively identifying smokers and offering smoking cessation support in primary care populations: A cluster‐randomized trial. *Addiction.* 2008;103(6):998-1006.

92. Myles P, Hendrata M, Layher Y, et al. Double-blind, randomized trial of cessation of smoking after audiotape suggestion during anaesthesia. *Br J Anaesth.* 1996;76(5):694-698.

93. Nagle AL, Hensley MJ, Schofield MJ, Koschel AJ. A randomised controlled trial to evaluate the efficacy of a nurse‐provided intervention for hospitalised smokers. *Aust N Z J Public Health.* 2005;29(3):285-291.

94. Nevid JS, Javier RA. Preliminary investigation of a culturally specific smoking cessation intervention for Hispanic smokers. *Am J Health Promot.* 1997;11(3):198-207.

95. Niaura R, Abrams DB, Shadel WG, Rohsenow DJ, Monti PM, Sirota AD. Cue exposure treatment for smoking relapse prevention: A controlled clinical trial. *Addiction.* 1999;94(5):685-695.

96. Okuyemi KS, Goldade K, Whembolua GL, et al. Motivational interviewing to enhance nicotine patch treatment for smoking cessation among homeless smokers: A randomized controlled trial. *Addiction.* 2013;108(6):1136-1144.

97. Okuyemi KS, James AS, Mayo MS, et al. Pathways to health: a cluster randomized trial of nicotine gum and motivational interviewing for smoking cessation in low-income housing. *Health Educ Behav.* 2007;34(1):43-54.

98. Ostroff JS, Burkhalter JE, Cinciripini PM, et al. Randomized trial of a presurgical scheduled reduced smoking intervention for patients newly diagnosed with cancer. *Health Psychol.* 2014;33(7):737-747.

99. Paek Y-J, Lee S, Kim Y-H, et al. Effect on smoking quit rate of telling smokers their health risk appraisal in terms of health age: A randomized control trial. *Asian Pac J Cancer Prev.* 2014;15:4963-4968.

100. Parkes G, Greenhalgh T, Griffin M, Dent R. Effect on smoking quit rate of telling patients their lung age: The Step2quit randomised controlled trial. *BMJ.* 2008;336(7644):598-600.

101. Patten CA, Martin JE, Myers MG, Calfas KJ, Williams CD. Effectiveness of cognitive-behavioral therapy for smokers with histories of alcohol dependence and depression. *J Stud Alcohol.* 1998;59(3):327-335.

102. Pbert L, Ockene JK, Zapka J, et al. A community health center smoking-cessation intervention for pregnant and postpartum women. *Am J Prev Med.* 2004;26(5):377-385.

103. Perkins KA, Marcus MD, Levine MD, et al. Cognitive–behavioral therapy to reduce weight concerns improves smoking cessation outcome in weight-concerned women. *J Consult Clin Psychol.* 2001;69(4):604-613.

104. Prapavessis H, Cameron L, Baldi JC, et al. The effects of exercise and nicotine replacement therapy on smoking rates in women. *Addict Behav.* 2007;32(7):1416-1432.

105. Prapavessis H, De Jesus S, Fitzgeorge L, Faulkner G, Maddison R, Batten S. Exercise to enhance smoking cessation: The getting physical on cigarette randomized control trial. *Ann Behav Med.* 2016;50(3):358-369.

106. Prochaska JJ, Hall SE, Delucchi K, Hall SM. Efficacy of initiating tobacco dependence treatment in inpatient psychiatry: A randomized controlled trial. *Am J Public Health.* 2014;104(8):1557-1565.

107. Prokhorov AV, Yost T, Mullin-Jones M, et al. “Look at your health”: Outcomes associated with a computer-assisted smoking cessation counseling intervention for community college students. *Addict Behav.* 2008;33(6):757-771.

108. Quist-Paulsen P, Gallefoss F. Randomised controlled trial of smoking cessation intervention after admission for coronary heart disease. *BMJ.* 2003;327(7426):1254-1247.

109. Ramos M, Ripoll J, Estrades T, et al. Effectiveness of intensive group and individual interventions for smoking cessation in primary health care settings: A randomized trial. *BMC Public Health.* 2010;10(1):89-94.

110. Ratner PA, Johnson JL, Richardson CG, et al. Efficacy of a smoking‐cessation intervention for elective‐surgical patients. *Res Nurs Health.* 2004;27(3):148-161.

111. Reid MS, Fallon B, Sonne S, et al. Smoking cessation treatment in community-based substance abuse rehabilitation programs. *J Subst Abuse Treat.* 2008;35(1):68-77.

112. Reitzel LR, McClure JB, Cofta-Woerpel L, et al. The efficacy of computer-delivered treatment for smoking cessation. *Cancer Epidemiology and Prevention Biomarkers.* 2011;20(7):1555-1557.

113. Rigotti NA, Park ER, Regan S, et al. Efficacy of telephone counseling for pregnant smokers: A randomized controlled trial. *Obstet Gynecol.* 2006;108(1):83-92.

114. Rigotti NA, Regan S, Levy DE, et al. Sustained care intervention and postdischarge smoking cessation among hospitalized adults: A randomized clinical trial. *JAMA.* 2014;312(7):719-728.

115. Rodondi N, Collet T-H, Nanchen D, et al. Impact of carotid plaque screening on smoking cessation and other cardiovascular risk factors: A randomized controlled trial. *Arch Intern Med.* 2012;172(4):344-352.

116. Rodríguez-Artalejo F, Urdinguio PL, Guallar-Castillón P, et al. One year effectiveness of an individualised smoking cessation intervention at the workplace: A randomised controlled trial. *Occup Environ Med.* 2003;60(5):358-363.

117. Rohsenow DJ, Martin RA, Monti PM, et al. Motivational interviewing versus brief advice for cigarette smokers in residential alcohol treatment. *J Subst Abuse Treat.* 2014;46(3):346-355.

118. Rohsenow DJ, Tidey JW, Martin RA, et al. Contingent vouchers and motivational interviewing for cigarette smokers in residential substance abuse treatment. *J Subst Abuse Treat.* 2015;55:29-38.

119. Romand R, Gourgou S, Sancho‐Garnier H. A randomized trial assessing the Five‐Day Plan for smoking cessation. *Addiction.* 2005;100(10):1546-1554.

120. Secades-Villa R, García-Rodríguez O, López-Núñez C, Alonso-Pérez F, Fernández-Hermida JR. Contingency management for smoking cessation among treatment-seeking patients in a community setting. *Drug Alcohol Depend.* 2014;140:63-68.

121. Siddiqi K, Khan A, Ahmad M, et al. Action to stop smoking in suspected tuberculosis (ASSIST) in Pakistan: A cluster randomized, controlled trial. *Ann Intern Med.* 2013;158(9):667-675.

122. Simmons VN, Heckman BW, Fink AC, Small BJ, Brandon TH. Efficacy of an experiential, dissonance-based smoking intervention for college students delivered via the Internet. *J Consult Clin Psychol.* 2013;81(5):810-820.

123. Simon JA, Carmody TP, Hudes ES, Snyder E, Murray J. Intensive smoking cessation counseling versus minimal counseling among hospitalized smokers treated with transdermal nicotine replacement: a randomized trial. *The American Journal of Medicine.* 2003;114(7):555-562.

124. Simon JA, Solkowitz SN, Carmody TP, Browner WS. Smoking cessation after surgery: A randomized trial. *Arch Intern Med.* 1997;157(12):1371-1376.

125. Smith PM, Corso L, Brown KS, Cameron R. Nurse case-managed tobacco cessation interventions for general hospital patients: Results of a randomized clinical trial. *Can J Nurs Res.* 2011;43(1):98-117.

126. Smith SS, Jorenby DE, Fiore MC, et al. Strike while the iron is hot: Can stepped-care treatments resurrect relapsing smokers? *J Consult Clin Psychol.* 2001;69(3):429-439.

127. Smits JA, Zvolensky MJ, Davis ML, et al. The efficacy of vigorous-intensity exercise as an aid to smoking cessation in adults with high anxiety sensitivity: A randomized controlled trial. *Psychosom Med.* 2016;78(3):354-364.

128. Stein MD, Weinstock MC, Herman DS, Anderson BJ, Anthony JL, Niaura R. A smoking cessation intervention for the methadone‐maintained. *Addiction.* 2006;101(4):599-607.

129. Stockings EA, Bowman JA, Baker AL, et al. Impact of a postdischarge smoking cessation intervention for smokers admitted to an inpatient psychiatric facility: A randomized controlled trial. *Nicotine Tob Res.* 2014;16(11):1417-1428.

130. Sykes CM, Marks DF. Effectiveness of a cognitive behaviour therapy self-help programme for smokers in London, UK. *Health Promotion International.* 2001;16(3):255-260.

131. Taylor CB, Miller NH, Herman S, et al. A nurse-managed smoking cessation program for hospitalized smokers. *Am J Public Health.* 1996;86(11):1557-1560.

132. Twardella D, Brenner H. Effects of practitioner education, practitioner payment and reimbursement of patients’ drug costs on smoking cessation in primary care: A cluster randomised trial. *Tob Control.* 2007;16(1):15-21.

133. Volpp KG, Troxel AB, Pauly MV, et al. A randomized, controlled trial of financial incentives for smoking cessation. *N Engl J Med.* 2009;360(7):699-709.

134. Wewers ME, Ferketich AK, Harness J, Paskett ED. Effectiveness of a nurse-managed, lay-led tobacco cessation intervention among Ohio Appalachian women. *Cancer Epidemiology and Prevention Biomarkers.* 2009;18(12):3451-3458.

135. Whiteley JA, Williams DM, Dunsiger S, et al. YMCA Commit to Quit. *Am J Prev Med.* 2012;43(3):256-262.

136. Wiggers LC, Smets EM, Oort FJ, et al. The effect of a minimal intervention strategy in addition to nicotine replacement therapy to support smoking cessation in cardiovascular outpatients: A randomized clinical trial. *Eur J Cardiovasc Prev Rehabil.* 2006;13(6):931-937.

137. Williams GC, Deci EL. Activating patients for smoking cessation through physician autonomy support. *Med Care.* 2001;39(8):813-823.

138. Wilson JS, Fitzsimons D, Bradbury I, Elborn JS. Does additional support by nurses enhance the effect of a brief smoking cessation intervention in people with moderate to severe chronic obstructive pulmonary disease? A randomised controlled trial. *Int J Nurs Stud.* 2008;45(4):508-517.

139. Winhusen TM, Brigham GS, Kropp F, Lindblad R, Gardin JG. A randomized trial of concurrent smoking-cessation and substance use disorder treatment in stimulant-dependent smokers. *The Journal of Clinical Psychiatry.* 2014;75(4):336-343.

140. Winickoff JP, Nabi-Burza E, Chang Y, et al. Sustainability of a parental tobacco control intervention in pediatric practice. *Pediatrics.* 2014;134(5):933-941.

141. Yalcin BM, Unal M, Pirdal H, Karahan TF. Effects of an anger management and stress control program on smoking cessation: A randomized controlled trial. *The Journal of the American Board of Family Medicine.* 2014;27(5):645-660.

142. Zheng P, Guo F, Chen Y, Fu Y, Ye T, Fu H. A randomized controlled trial of group intervention based on social cognitive theory for smoking cessation in China. *J Epidemiol.* 2007;17(5):147-155.

Exploratory and Sensitivity Analyses

Interactions Effects by Time and BCT Target on Smoking Cessation Rates

There was no significant interaction effect between time and BCTs on cessation rates either when the total smoking cessation BCTs were considered (*B* = -0.006, *p* = .396), or when personalised (*B* = -0.039, *p* = .084) and non-personalised (*B* = 0.001, *p* = .944) smoking cessation BCTs were considered separately. When the cluster robust re-estimation was used, there was evidence of a declining effect of personalised BCTs over time (*p* = .013), whereas results for the total number and non-personalised BCTs remained largely unchanged (*p* = .387, .937, respectively).

When examining the separate effects of quitting and abstinence BCTs within the same model, only the abstinence BCTs predicted smoking cessation rates (quitting: *B* = 0.003, *p* = .789; abstinence: *B* = 0.069, *p* = .025). As noted in the analysis plan, these two variables were highly correlated (Pearson’s *r* = .77, *p* < .001). When these were considered in separate models, both quitting (*B* = 0.024, *p* = .002) and abstinence (*B* = 0.075, *p* < .001) BCTs predicted smoking cessation rates. Finally, there was no evidence that quitting and abstinence BCTs interacted in their effect on smoking cessation rates (interaction term *B* = -0.002, *p* = .370).

Predicting Smoking Cessation while including all p < .1 Predictors

As described in the analysis plan, a sensitivity analysis adding all predictors demonstrating *p* < .1 into one model was pre-specified (pp. 9-10, <https://osf.io/24pzj/>). Results of this analysis are presented in Supplemental Table S1, below.

Supplemental Table B1

*Results of Sensitivity Analysis Including all Predictors Demonstrating p < .1 [B (SE)]*

|  | *B* (*SE*) | | |
| --- | --- | --- | --- |
| Received medication (1 = yes, 0 = no) | 0.213 | (0.159) |  |
| Personalised smoking cessation BCTs | -0.126 | (0.060) | * |
| Non-personalised smoking cessation BCTs | 0.007 | (0.009) |  |
| Mode of delivery (1 = person-delivered, 0 = written) | -0.425 | (0.306) |  |
| Personalised smoking cessation BCTs × mode of delivery | 0.165 | (0.063) | ** |
| Treatment engagement BCTs | 0.161 | (0.076) | * |

*Note*. BCT = behaviour change technique. Model was controlled for mean age, mean Fagerström Test for Nicotine Dependence, length of follow-up, cotinine verification, and point prevalence vs. sustained abstinence. Variables that formed part of interaction terms were not mean-centred. The ‘main’ effects of these variables should be interpreted as the effect of that variable when the other from the interaction term = 0. E.g., the ‘main’ effect of personalised BCTs is the simple effect when mode of delivery is written (mode of delivery = 0) and the ‘main’ effect of mode of delivery is the simple effect when no personalised BCTs were delivered (Personalised BCTs = 0). *** *p* < .001, ** *p* < .01, * *p* < .05, † *p* < .1.

Predicting Smoking Cessation while including All^a^ Studies and Controlling for Attrition

As described in the analysis plan, sensitivity analyses including all^a^ studies (not just the well-described ones) and including attrition were pre-specified (p. 10, <https://osf.io/24pzj/>). Results of these sensitivity analyses are presented in Supplemental Table S2, below.

Supplemental Table B2

*Results of Sensitivity Analyses Including All Studies and Including Attrition as a Covariate [B (SE)]*

| Model 1 | Primary Model | | | With All^a^ Studies Included | | | | | | With Attrition | | |
| --- | --- | --- | --- | --- | --- | --- | --- | --- | --- | --- | --- | --- |
| Received medication (1 = yes, 0 = no) | 0.334 | (0.155) | * | 0.301 | (0.149) | * | 0.264 | (0.149) | † | 0.321 | (0.154) | * |
| Total smoking cessation BCTs | 0.020 | (0.006) | *** | 0.016 | (0.006) | * | 0.124 | (0.067) | † | 0.020 | (0.006) | *** |
| Description quality |  |  |  | -0.009 | (0.084) |  | 0.085 | (0.101) |  |  |  |  |
| Total smoking cessation BCTs × description quality^b^ |  |  |  |  |  |  | -0.036 | (0.022) |  |  |  |  |
| Attrition |  |  |  |  |  |  |  |  |  | -1.353 | (0.405) | *** |
| Model 2 | Primary Model | | | With All^a^ Studies Included | | | | | | With Attrition | | |
| Received medication (1 = yes, 0 = no) | 0.346 | (0.156) | * | 0.316 | (0.149) | * | 0.274 | (0.149) | † | 0.335 | (0.155) | * |
| Personalised smoking cessation BCTs | 0.038 | (0.022) | † | 0.041 | (0.023) | † | 0.511 | (0.227) | * | 0.039 | (0.021) | † |
| Non-personalised smoking cessation BCTs | 0.016 | (0.008) | * | 0.010 | (0.008) |  | 0.065 | (0.074) |  | 0.016 | (0.008) | * |
| Description quality |  |  |  | -0.009 | (0.084) |  | 0.098 | (0.101) |  |  |  |  |
| Personalised smoking cessation BCTs × description quality^b^ |  |  |  |  |  |  | -0.160 | 0.077 | * |  |  |  |
| Non-personalised smoking cessation BCTs × description quality^b^ |  |  |  |  |  |  | -0.018 | 0.025 |  |  |  |  |
| Attrition |  |  |  |  |  |  |  |  |  | -1.357 | (0.404) | *** |
| Model 3 | Primary Model | | | With All^a^ Studies Included | | | | | | With Attrition | | |
| Received medication (1 = yes, 0 = no) | 0.266 | (0.166) |  | 0.228 | (0.162) |  | 0.188 | (0.162) |  | 0.271 | (0.165) |  |
| Total smoking cessation BCTs | -0.020 | (0.027) |  | -0.020 | (0.023) |  | 0.085 | (0.072) |  | -0.013 | (0.026) |  |
| Mode of delivery (1 = person-delivered, 0 = written) | -0.605 | (0.546) |  | -0.422 | (0.421) |  | -0.418 | (0.416) |  | -0.353 | (0.533) |  |
| Total smoking cessation BCTs × mode of delivery^b^ | 0.042 | (0.028) |  | 0.035 | (0.024) |  | 0.035 | (0.024) |  | 0.035 | (0.027) |  |
| Description quality |  |  |  | 0.020 | (0.095) |  | 0.122 | 0.115 |  |  |  |  |
| Total smoking cessation BCTs × description quality^b^ |  |  |  |  |  |  | -0.035 | 0.023 |  |  |  |  |
| Attrition |  |  |  |  |  |  |  |  |  | -1.337 | (0.443) | ** |
| Model 4 | Primary Model | | | With All^a^ Studies Included | | | | | | With Attrition | | |
| Received medication (1 = yes, 0 = no) | 0.231 | (0.164) |  | 0.213 | (0.161) |  | 0.182 | (0.161) |  | 0.257 | (0.166) |  |
| Personalised smoking cessation BCTs | -0.131 | (0.068) | † | -0.123 | (0.076) |  | 0.345 | (0.242) |  | -0.072 | (0.073) |  |
| Non-personalised smoking cessation BCTs | 0.031 | (0.038) |  | 0.019 | (0.035) |  | 0.064 | (0.083) |  | 0.012 | (0.039) |  |
| Mode of delivery (1 = person-delivered, 0 = written) | -0.235 | (0.564) |  | -0.243 | (0.436) |  | -0.367 | (0.435) |  | -0.183 | (0.565) |  |
| Personalised smoking cessation BCTs × mode of delivery^b^ | 0.186 | (0.071) | ** | 0.177 | (0.079) | * | 0.173 | (0.078) | * | 0.122 | (0.076) |  |
| Non-personalised smoking cessation BCTs × mode of delivery^b^ | -0.016 | (0.039) |  | -0.012 | (0.036) |  | -0.004 | (0.036) |  | 0.003 | (0.039) |  |
| Description quality |  |  |  | 0.013 | (0.093) |  | 0.125 | (0.112) |  |  |  |  |
| Personalised smoking cessation BCTs × description quality^b^ |  |  |  |  |  |  | -0.157 | (0.077) | * |  |  |  |
| Non-personalised smoking cessation BCTs × description quality^b^ |  |  |  |  |  |  | -0.017 | (0.025) |  |  |  |  |
| Attrition |  |  |  |  |  |  |  |  |  | -1.223 | (0.451) | ** |
| Model 5 | Primary Model | | | With All^a^ Studies Included | | | | | | With Attrition | | |
| Received medication (1 = yes, 0 = no) | 0.429 | (0.251) | † | 0.350 | (0.222) |  | 0.254 | (0.226) |  | 0.390 | (0.244) |  |
| Total smoking cessation BCTs | 0.010 | (0.008) |  | 0.005 | (0.009) |  | 0.128 | (0.067) | † | 0.013 | (0.008) |  |
| Treatment engagement BCTs | 0.176 | (0.079) | * | 0.181 | (0.086) | * | 0.196 | (0.086) | * | 0.185 | (0.078) | * |
| Medication adherence BCTs | 0.044 | (0.078) |  | 0.043 | (0.084) |  | 0.042 | (0.083) |  | -0.026 | (0.090) |  |
| Medication adherence BCTs × received medication^b^ | -0.067 | (0.089) |  | -0.054 | (0.092) |  | -0.035 | (0.092) |  | -0.007 | (0.099) |  |
| Description quality |  |  |  | -0.012 | (0.083) |  | 0.095 | (0.100) |  |  |  |  |
| Total smoking cessation BCTs × description quality^b^ |  |  |  |  |  |  | -0.042 | (0.023) | † |  |  |  |
| Attrition |  |  |  |  |  |  |  |  |  | -1.312 | (0.398) | *** |
| Model 6 | Primary Model | | | With All^a^ Studies Included | | | | | | With Attrition | | |
| Received medication (1 = yes, 0 = no) | 0.399 | (0.260) |  | 0.309 | (0.227) |  | 0.224 | (0.230) |  | 0.356 | (0.252) |  |
| Personalised smoking cessation BCTs | 0.021 | (0.023) |  | 0.026 | (0.025) |  | 0.525 | (0.224) | * | 0.024 | (0.023) |  |
| Non-personalised smoking cessation BCTs | 0.007 | (0.011) |  | -0.001 | (0.011) |  | 0.069 | (0.074) |  | 0.010 | (0.010) |  |
| Treatment engagement BCTs | 0.171 | (0.080) | * | 0.171 | (0.087) | * | 0.191 | (0.086) | * | 0.179 | (0.079) | * |
| Medication adherence BCTs | 0.046 | (0.078) |  | 0.048 | (0.085) |  | 0.048 | (0.083) |  | -0.026 | (0.091) |  |
| Medication adherence BCTs × received medication^b^ | -0.057 | (0.092) |  | -0.039 | (0.094) |  | -0.026 | (0.093) |  | 0.005 | (0.102) |  |
| Description quality |  |  |  | -0.013 | (0.083) |  | 0.108 | (0.100) |  |  |  |  |
| Personalised smoking cessation BCTs × description quality^b^ |  |  |  |  |  |  | -0.169 | (0.076) | * |  |  |  |
| Non-personalised smoking cessation BCTs × description quality^b^ |  |  |  |  |  |  | -0.023 | (0.025) |  |  |  |  |
| Attrition |  |  |  |  |  |  |  |  |  | -1.319 | (0.400) | *** |

*Note*. BCT = behaviour change technique. All models were controlled for mean age, mean Fagerström Test for Nicotine Dependence, length of follow-up, cotinine verification, and point prevalence vs. sustained abstinence. *** *p* < .001, ** *p* < .01, * *p* < .05, † *p* < .1.

a. All comparator groups were included regardless of the quality of their BCT descriptions; however, seven groups still had to be excluded due to missing data on the control variables. b. Variables that formed part of interaction terms were not mean-centred. The ‘main’ effects of these variables should be interpreted as the effect of that variable when the other from the interaction term = 0. E.g., in Model 3, the ‘main’ effect of BCTs is the simple effect when mode of delivery is written (mode of delivery = 0) and the ‘main’ effect of mode of delivery is the simple effect when no BCTs were delivered (BCTs = 0).

Interactions Effects by Smoking Cessation BCTs and Receipt of Medication on Smoking Cessation Rates

During the peer review process, one reviewer raised the interesting suggestion of whether the impacts of smoking cessation BCTs and medication on smoking cessation can be considered independently, or whether they interact with one another to influence smoking cessation. To investigate this, we conducted unplanned exploratory analyses to test these interactions. We found no evidence that receipt of medication interacted with the total number of smoking cessation BCTs (*B* = 0.000, *p* = .981), the number of personalised smoking cessation BCTs (*B* = 0.009, *p* = .839), or the number of non-personalised smoking cessation BCTs (*B* = -0.001, *p* = .942).
